# Aging and Hypertension – Independent or Intertwined White Matter Impairing Factors? Insights From the Quantitative Diffusion Tensor Imaging

**DOI:** 10.3389/fnagi.2019.00035

**Published:** 2019-02-19

**Authors:** Agnieszka Sabisz, Patrycja Naumczyk, Anna Marcinkowska, Beata Graff, Dariusz Gąsecki, Anna Glińska, Marta Witkowska, Anna Jankowska, Aleksandra Konarzewska, Jerzy Kwela, Krzysztof Jodzio, Edyta Szurowska, Krzysztof Narkiewicz

**Affiliations:** ^1^Second Department of Radiology, Medical University of Gdańsk, Gdańsk, Poland; ^2^Institute of Psychology, University of Gdańsk, Gdańsk, Poland; ^3^Department of Hypertension and Diabetology, Medical University of Gdańsk, Gdańsk, Poland; ^4^Department of Neurology of Adults, Medical University of Gdańsk, Gdańsk, Poland; ^5^Institute of Experimental Physics, University of Gdańsk, Gdańsk, Poland

**Keywords:** DTI, aging, hypertension, quantitative fiber tracking, diffusion tensor imaging

## Abstract

Aging disrupts white matter integrity, and so does continuous elevated blood pressure that accompanies hypertension (HTN). Yet, our understanding of the interrelationship between these factors is still limited. The study aimed at evaluating patterns of changes in diffusion parameters (as assessed by quantitative diffusion fiber tracking – qDTI) following both aging, and hypertension, as well as the nature of their linkage. 146 participants took part in the study: the control group (*N* = 61) and the patients with hypertension (*N* = 85), and were divided into three age subgroups (25–47, 48–56, 57–71 years). qDTI was used to calculate the values of fractional anisotropy, mean, radial and axial diffusivity in 20 main tracts of the brain. The effects of factors (aging and hypertension) on diffusion parameters of tracts were tested with a two-way ANOVA. In the right hemisphere there was no clear effect of the HTN, nor an interaction between the factors, though some age-related effects were observed. Contrary, in the left hemisphere both aging and hypertension contributed to the white matter decline, following a functional pattern. In the projection pathways and the fornix, HTN and aging played part independent of each other, whereas in association fibers and the corpus callosum if the hypertension effect was significant, an interaction was observed. HTN patients manifested faster decline of diffusion parameters but also reached a plateau earlier, with highest between-group differences noted in the middle-aged subgroup. Healthy and hypertensive participants have different brain aging patterns. The HTN is associated with acceleration of white matter integrity decline, observed mainly in association fibers of the left hemisphere.

## Introduction

A novel magnetic resonance imaging (MRI) method that allows an exploration of the white matter (WM) microstructure integrity is the diffusion tensor imaging (DTI). In DTI sequence, the intensity of each image element (voxel) expresses the rate of the water random walk (diffusion) at that location in one of the gradients directions (for reviews Jones, 2008; Tournier et al., 2011). Fitting the diffusion tensor model to the diffusion MRI signal enables computation of the diffusion coefficients. These are: the fractional anisotropy (FA), which is a marker of directionality; the mean diffusivity (MD), which defines general properties of tissue; the axial diffusivity (AD), which is an index of axonal injury and the radial diffusivity (RD), which is associated with the myelin degeneration (Song et al., 2003, 2005; Winklewski et al., 2018). The diffusion coefficients can be further contrasted between the populations of interest. Typically, the comparison is being made at a whole-brain level, but it can also be restricted to specific brain WM pathways. The latter method, referred to as a quantitative fiber tracking, stands as a promising tool for examining WM damage as it increases the specificity of the assessment (Sullivan et al., 2010b).

The integrity of the WM, as measured by the diffusion coefficients, is affected by the physiological aging of the brain. It declines proportionally with age, meaning that the more advanced the age, the lower the fractional anisotropy, and the higher the diffusivity parameters. The process starts at the age of 20 (Sullivan et al., 2010a,b) and continues throughout adulthood irrespective of one’s sex (Sullivan et al., 2001). The advanced rate of these changes differs throughout the brain. The anterior–posterior gradient of the FA decrease and MD, RA, AD increase has been observed, where the anterior areas progress earlier than the posterior ones (Salat et al., 2005; Bucur et al., 2008; Bennett et al., 2010). This spatial gradient was primarily explored within the corpus callosum (Hasan et al., 2010; Michielse et al., 2010; Lebel et al., 2012; Voineskos et al., 2012; Bennett et al., 2017) and it was proven to represent a gradual deterioration rather than simply frontal lobes impairment (Davis et al., 2009).

Additionally, a superior–inferior gradient of the age-related WM integrity decline has been proposed as well (Sullivan et al., 2010b,c). The superior WM tracts are supposed to demonstrate faster deterioration with aging than the inferior pathways (Michielse et al., 2010; Mårtensson et al., 2017). However, this spatial gradient may be interpreted also as a functional one. Numerous studies have proven a differential progression rate of the WM integrity impairment for the projection fibers when compared with the association ones (Stadlbauer et al., 2008; Hasan et al., 2010; Lebel et al., 2012; Voineskos et al., 2012; Bender and Raz, 2015; Bender et al., 2016). As most of the projection fibers are located inferiorly to association ones – this functional diversity may be the basis of the postulated superior-inferior spatial gradient. Furthermore, an interhemispheric sensitivity to WM deterioration was investigated as well with somewhat ambiguous results. Some studies have proven major variability of age-related changes in diffusivity parameters between the corresponding tracts of the hemispheres (Zahr et al., 2009; Hasan et al., 2010; Wahl et al., 2010), some reported only mild effects, constrained to a few fibers effects (Michielse et al., 2010; Lebel et al., 2012), and some did not discover any hemisphere-specific WM aging patterns at all (Stadlbauer et al., 2008; Voineskos et al., 2012).

The aforementioned patterns of age-related macrostructural WM degenerations are observed in individuals classified as ones aging successfully. However, aging itself raises risks of many additional diseases that also contribute to brain tissue damage, out of which the vascular impairing factors, such as hypertension, are of special interest (Scott et al., 2015). Previous magnetic resonance imaging (MRI) studies of the hypertensive patients uncovered both gray matter and white matter volumetric abnormalities in the group. Cortical areas that manifested the highest vulnerability to the heightened blood pressure included the prefrontal cortex (Raz et al., 2007a), the hippocampus (den Heijer et al., 2005; Raz et al., 2005) and the inferior temporal cortex (Taki et al., 2004), all of which are prone to age-related damage. Hypertension was also shown to affect other brain regions moderately sensitive to aging, such as the additional motor areas (Chen et al., 2006), the cuneus (Taki et al., 2004), and the thalamus (Strassburger et al., 1997). Also, in the study population, the macrostructural damage of the WM has been demonstrated through magnified occurrence rates of the white matter hyperintensities (WMH) (Raz et al., 2007b; Shrestha et al., 2009; Dickie et al., 2016).

In contrast to the above-mentioned comprehensive articles, the WM microstructure (integrity) disruption associated with hypertension has been scarcely reported. DTI studies of the patients revealed abnormalities regarding some of the tracts, mostly in association fibers and the corpus callosum (Gons et al., 2012; Maillard et al., 2012; Mcevoy et al., 2015). What is prominent is that the pattern of those differences followed the one typically attributed to normal aging; that is, the hypertensive patients’ FA was significantly decreased compared with peers. Also, the systolic blood pressure (SBP) was linearly associated with decreased regional FA and increased MD, especially in the anterior corpus callosum, the inferior fronto-occipital fasciculi and the fibers that project from the thalamus to the superior frontal gyrus (Maillard et al., 2012; Mcevoy et al., 2015). Also, hypertension was associated with FA decline and MD, AD, RD growth in frontal brain regions (Kennedy and Raz, 2009). It is notable, that this pattern reflects the anterior–posterior gradient of WM aging cited earlier.

The overview provided above highlights an overlap between the patterns of WM integrity damage following normal aging and the one associated with hypertension. What is startling is that no previous study has explored both these variables at the same time using quantitative fiber tracking. Even the big cohort studies, such as Framingham study (Maillard et al., 2012) or Rotterdam study (de Groot et al., 2016) only compared the vascular risks between the groups adjusted for age (meaning the variable influence was regressed from the assessment). Additionally, the studies did not differentiate between tracts of the respective hemispheres, lacking the information on possible right-to-left differences in the groups. Therefore, the relationship among WM damage, aging and hypertension is still not fully understood. Is the HTN’s contribution to WM integrity disruption independent of age? Or do those variables interact? What is the spatial distribution of this relationship? Does it affect all tracts equally? Are there any spatial or functional gradients observed? These were the questions we addressed in our study. We performed quantitative fiber tracking in hypertensive patients and controls in three age-matched groups comparing four diffusion coefficients (FA, MD, AD, RD) in nine hemisphere-specific tracts, fornix and corpus callosum. Our focus was on the relationship between hypertension’s and the age’s interaction in subjects of different age groups.

## Materials and Methods

### Participants

A 146 participants took part in the study: the control group (CON) of 61 healthy subjects (mean age: 50 ± 11 years, range: 25–71) and the patient’s group (HTN) of 85 hypertensive individuals (mean age: 53 ± 10 years, range: 22–78). The exclusion criteria for the study included: coronary heart disease, heart failure, lung diseases, kidney failure, cancer, diabetes, previous stroke or TIA, severe head trauma, meningitis, psychiatric disorders, addiction (medications, alcohol, narcotics), claustrophobia, or existing metal implants. Also, all subjects underwent thorough neuropsychological examination (on the day of the RMI scan), which confirmed cognitive norm status. Detailed results of this assessment exceed the scope of the present paper and therefore will not be discussed further. The study protocol was approved by the Ethics Committee of the Medical University of Gdańsk (NKEBN/422/2011). All participants were informed about the study merits and signed a written consent.

Due to the wide range of the participants’ ages, three age-related subgroups were determined, resulting overall with six subgroups for comparison (HTN × age). The age margins were chosen to maximize the equipotency of subjects’ quantity in each subgroup [group I (young group) – 25–47 years, group II (middle-aged group)- 48–56 years, group III (older group) – 57–71 years]. The differences between thus defined “HTN × age” subgroups’ subject numbers were insignificant (chi-square test, *p* > 0.1), nor between the age of the corresponding CON and HTN groups (group I – *t* = −0.567, *p* > 0.1, group II – *t* = 0.522, *p* > 0.1, group III – *t* = −0.739, *p* > 0.1). Basic characteristics of the groups are provided in Table 1.

**Table 1:** Characteristics of the groups.

	Group I (25–47 years)		Group II (48–56 years)		Group III (57–71 years)	
	CON	HTN	CON	HTN	CON	HTN
*N*	22	24	19	29	20	32
Mean age (years)	39 ± 8	40 ± 7	53 ± 3	52 ± 3	62 ± 4	62 ± 4
BMI (kg/m^2^)	26.3 ± 3.9	28.5 ± 4.8	26.5 ± 3.2	29.5 ± 4.8	27.0 ± 4.2	29.9 ± 3.4
WHR	0.88 ± 0.07	0.93 ± 0.08	0.90 ± 0.08	0.94 ± 0.08	0.91 ± 0.06	0.95 ± 0.09
SBP 24 h (mm Hg)	116 ± 7	129 ± 9	117 ± 5	128 ± 11	117 ± 7	127 ± 11
DBP 24 h (mm Hg)	73 ± 5	80 ± 8	75 ± 5	80 ± 9	74 ± 5	76 ± 6
BP 24 h	75 ± 9	73 ± 8	73 ± 9	74 ± 7	74 ± 9	71 ± 9
HTN duration (years)	0	8 (range 0–24)	0	10 (range 0–30)	0	13 (range 0–52)
Treatment:						
Antihypertensive therapy	0	58%	0	76%	0	88%
Number of antihypertensive drugs Median (range)	0	1 (0–4)	0	2 (0–6)	0	2 (0–6)
ACE inhibitors	0	38%	0	46%	0	53%
Angiotensin II receptor antagonists	0	13%	0	29%	0	25%
Calcium channel blockers	0	17%	0	29%	0	41%
Diuretics	0	21%	0	28%	0	34%
β-Blockers	0	17%	0	46%	0	38%
α-Blockers	0	0%	0	11%	0	19%
Fazekas scale						
0	19	22	13	14	10	19
1	3	2	6	13	8	9
2	0	0	0	2	2	4
3	0	0	0	0	0	0

The between-group differences regarding body mass index (BMI), and waist-to-hip ratio (WHR) were evaluated with Mann–Whitney *U*-test (CON vs. HTN in each age subgroup) and Kruskal–Wallis *H*-test (age between-group comparison in CON/HTN subgroups). The analyses were performed with non-parametric tests because the data did not meet the assumption of normal distribution. All statistical tests were performed on IBM SPSS, version 24 (IBM, Corp., Armonk, NY, United States) with *p* < 0.05 being considered a statistically significant effect.

### Medication and Blood Pressure Measures

The diagnosis of hypertension was based on the 2013 European Society of Hypertension/European Society of Cardiology criteria. Twenty-four-hour ambulatory blood pressure monitoring (ABPM) was used in every subject to confirm blood pressure status. ABPM was performed within 3 weeks following a functional MRI study with the Spacelabs 90207 recorder (Spacelabs, Inc.). The recorders were programmed to obtain measurements every 20 min from 6:00 AM to 10:00 PM (day), and every 30 min from 10:00 PM to 6:00 AM (night). The results of the ABPM are presented in Table 1. In subjects who were not previously diagnosed with hypertension and patients with the history of hypertension who did not receive pharmacological treatment ABPM was used to confirm the blood pressure status. Average day-time SBP of ≥135 mmHg defined hypertension. Patients with the history of hypertension receiving blood pressure lowering drugs were not re-classified according to ABPM. Blood pressure results were contrasted analogously to BMI and WHR between the six age/HTN subgroups (see section “Participants”). The average hypertension duration ranged from 0 to 52 years (mean: 4.7 ± 6.9 years). The hypertension duration was determined in medical interview by the patient himself, and as such should be taken with caution of being not fully definitive. Mean number of antihypertensive drugs was 1.74 (median value 2.0), detailed in Table 1. Lipid-lowering drugs were used in 35% of patients and 15% of controls.

### DTI Acquisition

Magnetic resonance imaging examinations were performed on Philips Achieva 3.0T TX scanner (Philips Healthcare, Best, Netherlands) with the 32-channel head coil. DTI echo planar imaging sequence involved *b*-values of 0 and 800 s/mm^2^ with 32 gradient directions (TR = 6900 ms, TE = 65 ms, voxel size: 2 mm × 2 mm × 2 mm, FOV: 230 mm × 230 mm, 70 slices, NSA = 1). The scan orientation was axial without angulation. FLAIR sequence was scanned in transverse direction to AC-PC and acquisition parameters was: TR = 11000 ms, TE = 125 ms, TI = 2800 ms, voxel size: 1 mm × 1 mm × 4 mm, gap: 1 mm, FOV: 250 mm × 250 mm, slices 34, NSA = 1.

### White Matter Hyperintensities Evaluation

Magnetic resonance imaging assessments included ratings of white matter lesions based on FLAIR sequence. Changes in the white matter (features of leukoaraiosis) were classified according to the Fazekas scale by two independent, experienced radiologists (Fazekas et al., 1987, 1993). The scale is a four-level one, where the range of hyperintense white matter changes in the paraventricular and subcortical areas of the brain varies from 0 to 3, where 0 means no white matter lesions, 1 – single changes, 2 – numerous changes, 3 – confluent areas of WMH. The agreement between observers was compared based on the interrater reliability analyses using Cohen’s kappa coefficient. Between group difference was calculated with Mann–Whitney *U*-test (CON vs. HTN in each age subgroup) and Kruskal–Wallis *H*-test (age between-group comparison in CON/HTN subgroups).

### DTI Preprocessing

The data was processed in fully automatic pipeline. DTIs were first analyzed on the ExploreDTI software (Leemans et al., 2009). The data were converted to ^∗^.mat, then estimated to tensor model by the REKINDLE algorithm. Images were corrected for movement and eddy currents distortions, also the B matrix was accordingly rotated (Leemans and Jones, 2009). The data in each step were checked for the presence of artifacts. Residuals and outliers’ profiles were inspected. Participants whose motion exceeded 0.5 mm or 0.5°, had artifacts occurring, or whose outlier profile was greater than 10%, would have been disqualified from further analyses. No subjects met these criteria.

Then DTI images with gradients tables (after correction) and *b*-values were generated and used in subsequent analyses. The non-brain tissue was extracted from images using BET FSL, with a fractional intensity threshold of 0.2. The data were used to generate diffusion parameters’ maps (FA, MD, RD, AD) in the DTIFIT FSL (Behrens et al., 2003). During the next steps, FA images of all patients were aligned to the standard FA image, with a resolution of 1 mm × 1 mm × 1 mm (FMRIB58_FA) using the FNIRT non-linear registration tool (?). A mean FA image and mean FA skeleton was created as well. Information about the non-linear transformation and the FA skeleton was included in the averaging and the creation of each subject’s aligned MD, AD, and RD data.

### Quantitative DTI Statistics

Masks of the whole brain, the whole white matter, the left hemisphere and the right hemisphere and white matter of each of the hemisphere’s were prepared in fslmaths (Jenkinson et al., 2012). On their basis, average values of FA, MD, AD, RD for each subject/each mask were calculated. Additionally, probabilistic masks of white matter tractography (John Hopkins University white matter tractography atlas) were used to plot tracts of WM in the FSL (Oishi et al., 2008). Masks were converted to zero – one values, where 0 meant areas outside the route, and 1 – the areas which included the fiber of interest. Then, prepared masks were applied to the images of FA, MD, RD, and AD for each participant. In the last step mean values of the parameters of diffusion in all projections were calculated.

Nine, hemisphere-specific (two projection fibers and seven association fibers), and two commissural tracts were identified resulting in overall 20 pathways in the final analyses [L – left, R – right: Anterior thalamic radiation L/R, Corticospinal tract L/R, Cingulum (hippocampus) L/R, Cingulum (cingulate gyrus) L/R, Inferior fronto-occipital fasciculus L/R, Inferior longitudinal fasciculus L/R, Superior longitudinal fasciculus L/R, Superior longitudinal fasciculus (temporal part) L/R, Uncinate fasciculus L/R, Corpus callosum, Fornix]. Masks of the tracts superimposed on the brain are shown on figures in Supplementary Materials (SM_Figures_all.pdf).

As there are some papers suggesting noxious effect of one’s weight on WM diffusivity parameters (Stanek et al., 2011; Xu et al., 2013; Allen et al., 2016), we tested for correlations between both the BMI and the WHR, and the calculated diffusivity scores, to determine if including these covariates in the model is a necessity. None of the comparisons met the criterion of reasonable correlation (r coefficient ranging from 0.3 to 0.9), therefore the weight’s measures were not included in the final analyses. All of the correlation coefficients are presented in the Supplementary Material in Table SM1. The effects of age and hypertension on diffusion parameters were tested with a two-way ANOVA (disease: CON/HTN and age: group I – 25–47 years, group II – 48–56 years, group III – 57–71 years as factors). If the interaction between the factors appeared significant, the *post hoc* contrasts with the Bonfferoni correction for the multiple comparisons were calculated to determine the simple effects’ directions. Likewise, for the demographic analyses, the ANOVA was performed in IBM SPSS package, version 24, with *p* < 0.05 significance threshold.

## Results

### Demographic and Medical Data

Demographic characteristics of the groups divided into age subgroups are presented in Table 1.

No differences were found in BMI of subsequent HTN/CON age subgroups [CON: *H*(2) = 0.163, *p* > 0.1, HTN: *H*(2) = 3.834, *p* > 0.1], neither between the patients and controls in the young age subgroup (*U* = 195, *p* > 0.1). Significant differences were noted when comparing the HTN and CON groups in the middle-aged (*U* = 158, *p* = 0.034) and older (*U* = 176, *p* = 0.005) subgroups. Yet, it should be noted that the median and mean BMI within the groups of both the CON and the HTN fell into the same range (25–30) interpreted as overweight in National Institute of Health scale.

Analysis of WHR found no significant differences in all of the comparisons [CON age between-group comparison: *H*(2) = 1.550, *p* > 0.1, HTN age between-group comparison: *H*(2) = 3.834, *p* > 0.1, CON vs. HTN young subgroup: *U* = 178, *p* = 0.059, middle-aged subgroup: *U* = 183, *p* > 0.1, older subgroup: *U* = 219, *p* = 0.071].

In SBP level HTN/CON the age subgroups were similar [CON: *H*(2) = 0.001, *p* > 0.1, SUB: *H*(2) = 1.532, *p* > 0.1]. Concurrently, all HTN vs. CON contrasts revealed higher SBP in the HTN subgroups (young subgroup: *U* = 72, *p* < 0.001, middle-aged subgroup: *U* = 98, *p* = 0.001, older subgroup: *U* = 166, *p* = 0.003).

No differences in DBP level were found between successive CON age subgroups [H(2) = 1.823, *p* > 0.1]. In the HTN subgroup, the older group had significantly lower DBP than the younger groups [*H*(2) = 7.366, *p* = 0.025]. The HTN vs. CON comparisons revealed higher DBP level in the young and middle-aged subgroups (*U* = 116, *p* = 0.001 and *U* = 140, *p* = 0.016, respectively), but not in the older group (*U* = 264, *p* > 0.1).

### WMH Evaluation

The Fazekas scale’s scores of each of the subgroups are presented in Table 1. The estimated kappa coefficient was κ = 0.667 (*p* < < 0.001), which is considered substantial agreement between the observers. There were no significant intergroup differences (HTN vs. CON) in WMH burden (Mann–Whitney *U*: young subgroup: *U* = 250, *p* > 0.1, middle-aged subgroup: *U* = 214, *p* > 0.1, older subgroup: *U* = 297, *p* > 0.1). Yet, in both groups (HTN and CON) the number of WMH increased with age [CON: *H*(2) = 6.995, *p* = 0.031, SUB: *H*(2) = 11.099, *p* = 0.004].

### DTI Data

The detailed results for all diffusion coefficients and all tissues are provided in the Supplementary Material. Here we sum up the general patterns discovered. The two-way ANOVA revealed that both age and hypertension contributed to the differences noted in the study population, reflecting a decrease of the FA and an increase of all the other diffusivity parameters (MD, RD, AD). Yet some specific variations were observed as well, regarding the fiber’s functionality, the cerebral hemisphere asymmetry, as well as the diffusion coefficient being compared. Significant results are summarized in Table 2 (main effects and interaction effects) and Table 3 (simple effects).

**Table 2:** A schematic presentation of the results of the two-way ANOVA in the study population (age and disease as factors) grouped by type of fibers: blue – projection pathways, orange – commissural pathways, green – fornix, pink – associative pathways.

	*FA*	*MD*	*RD*	*AD*
Whole brain	**-**	**-**	**-**	**-**
Whole WM	**-**	**A**	**A**	**-**
LH brain	**-**	**-**	**-**	**-**
LH WM	**-**	**-**	**-**	**-**
RH brain	**-**	**-**	**-**	**-**
RH WM	**-**	**A**	**A**	**A**
L1	**A**	**A, H**	**A, H**	**A, H**
L2	**A**	**A**	**A**	**H**
R1	**A**	**A**	**A**	**A**
R2	**A**	**H**	**-**	**H**
FX	**A**	**A, H**	**A**	**A, H**
CC	**A**	***i***	***i***	***i***
L3	**A**	***i***	***i***	**A**
L4	**A**	**H**	**A**	**H**
L5	**A**	***i***	***i***	**A**
L6	**A**	***i***	***i***	***i***
L7	**A**	**A**	**A**	**A**
L8	**A**	**A**	**A**	**A**
L9	**A**	***i***	***i***	***i***
R3	-	**-**	**A**	**-**
R4	**A**	-	-	-
R5	**A**	**A**	**A**	**-**
R6	**A**	**-**	**A**	**-**
R7	**A**	**A**	**A**	**A**
R8	**A**	**A**	**A**	**-**
R9	**-**	**A, H**	**A, H**	**A, H**

*An “A” mark represents a significant main effect (*p* < 0.05) of the age of a respective tract. An “H” mark represents a significant main effect (*p* < 0.05) of the disease for a respective tract. An “i” mark represents if the interaction is present, simple effects are itemized in table YY. FA, fractional anisotropy; MD, mean diffusivity; RD, radial diffusivity; AD, axial diffusivity; WM, white matter; LH, left hemisphere; RH, right hemisphere; L1, anterior thalamic radiation L; R1, anterior thalamic radiation R; L2, corticospinal tract L; R2, corticospinal tract R; L3, cingulum (hippocampus) L; R3, cingulum (hippocampus) R; L4, cingulum (cingulate gyrus) L; R4, cingulum (cingulate gyrus) R; L5, inferior fronto-occipital fasciculus L; R5, inferior fronto-occipital fasciculus R; L6, inferior longitudinal fasciculus L; R6, inferior longitudinal fasciculus R; L7, superior longitudinal fasciculus L; R7, superior longitudinal fasciculus R; L8, superior longitudinal fasciculus (temporal part) L; R8, superior longitudinal fasciculus (temporal part) R; L9, uncinate fasciculus L; R9, uncinate fasciculus R; CC, corpus callosum; FX, fornix.*

**Table 3:** A schematic presentation of the results of simple effects analysis of age and disease factors in tracts where interaction is present in the study population.

			CC			L3		L5		L6			L9		
			MD	RD	AD	MD	RD	MD	RD	MD	RD	AD	MD	RD	AD
Group I		CON-HTN			+										
Group II		CON-HTN	+	+	+			+	+	+			+	+	+
Group III		CON-HTN													
CON		Group I – group II													
		Group I – group III	+	+	+			+	+	+	+	+	+	+	+
		Group II – group III	+	+	+	+	+	+	+	+	+	+	+	+	+
HTN		Group I – group II	+	+			+	+	+	+	+		+	+	
		Group I – group III		+		+	+	+	+	+	+	+	+	+	
		Group II – group III													

*Significant simple effects (p < 0.05) of the age groups (I, II, III) and disease (CON, HTN) factor is itemized and marked as “+.” FA, fractional anisotropy; MD, mean diffusivity; RD, radial diffusivity; AD, axial diffusivity. Age group I – 25–47 years old, II – 48–56 years old, III 57–71 years old. CC, corpus callosum; L3, cingulum (hippocampus) L; L5, inferior fronto-occipital fasciculus L; L6, inferior longitudinal fasciculus L; L9, uncinate fasciculus.*

**FIGURE 1:**
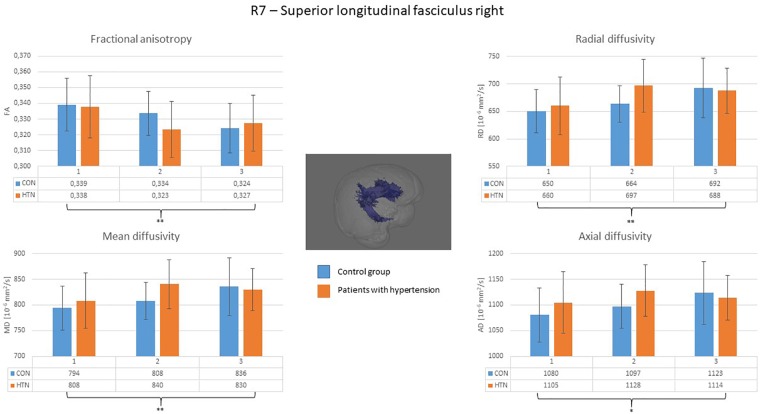
Diffusion coefficients of the right superior longitudinal fasciculus in all subgroups. The main effect of the age was significant for all of the diffusion coefficients. CON, the control group; HTN, the hypertensive group; 1, age subgroup of 25–47 years; 2, age subgroup of 48–56 years; 3, age subgroup of 57–71 years; FA, fractional anisotropy; MD, mean diffusivity; RD, radial diffusivity; AD, axial diffusivity. ^∗^*p* < 0.05, ^∗∗^*p* < 0.01, and ^∗∗∗^*p* < 0.001.

**FIGURE 2:**
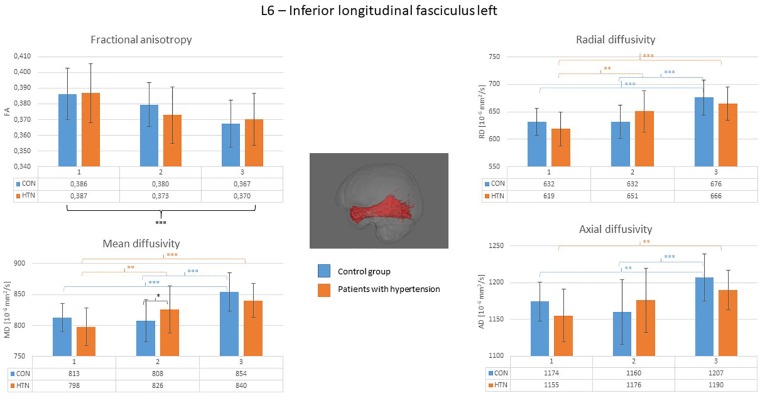
Diffusion coefficients of the left inferior longitudinal fasciculus in all subgroups. For the fractional anisotropy only the main effect of age was observed. For the other diffusion coefficients, the interaction of the age × hypertension was observed with the biggest intergroup difference apparent in the middle-aged group. CON, the control group; HTN, the hypertensive group; 1, age subgroup of 25–47 years; 2, age subgroup of 48–56 years; 3, age subgroup of 57–71 years; FA, fractional anisotropy; MD, mean diffusivity; RD, radial diffusivity; AD, axial diffusivity. ^∗^*p* < 0.05, ^∗∗^*p* < 0.01, and ^∗∗∗^*p* < 0.001.

**FIGURE 3:**
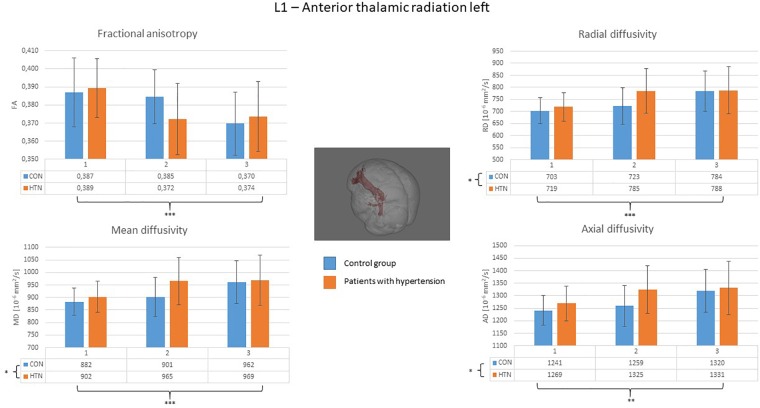
Diffusion coefficients of the left anterior thalamic radiation in all subgroups. For the fractional anisotropy only the main effect of the age was significant, whereas the independent effect of the age and the hypertension was significant for all of the other diffusion coefficients. CON, the control group; HTN, the hypertensive group; 1, age subgroup of 25–47 years; 2, age subgroup of 48–56 years; 3, age subgroup of 57–71 years; FA, fractional anisotropy; MD, mean diffusivity; RD, radial diffusivity; AD, axial diffusivity. ^∗^*p* < 0.05, ^∗∗^*p* < 0.01, and ^∗∗∗^*p* < 0.001.

A clear right to left hemisphere alteration was observed, where the right hemisphere’s white matter tissue and tracts were sensitive predominantly to age-related damage only (as shown on example of the right superior longitudinal fasciculus on Figure 1), whereas the left hemisphere’s tracts were more likely to present an interaction/independent contribution of both hypertension and the age (the interaction effect is shown on example of the left inferior longitudinal fasciculus on Figure 2, the independent contribution is shown on example of the left anterior thalamic radiation on Figure 3). There were only two tracts that fell short of this pattern – the right corticospinal tract (which presented a significant main effect of hypertension for MD and AD) and the right uncinate fasciculus (which demonstrated significant independent main effects of both hypertension and age for MD, AD, and RD).

Additionally, the impact of the age and the disease on projection, association and commissural pathways was differential. In projection pathways and in the fornix the factors tended to impair the white matter independent of each other, whereas in association fibers and the corpus callosum if hypertension contributed to the WM damage, an interaction of the factors was present. Two association fibers did not follow this pattern – left cingulum at the cingulate gyrus section (in which AD and MD showed significant independent main effects of the fibers) and again the right uncinate fasciculus.

The simple effects analyses confirmed, that it was the middle-age group that mostly contributed to the interaction effect observed. The HTN’s middle-age group had significantly lower FA values and higher diffusion coefficients than the middle-age CON subjects. Also, in the HTN group there was no difference between the middle-aged and older patients (with preserved significant differences between the young and both other groups), while in the CON group this pattern was observed earlier (no difference between the young and the middle-aged participants, with significant differences between the older group and both of the other ones). In other words, in the CON group, the differences between the consecutive age groups roughly followed an exponential function outline, whereas in the HTN group – a logarithm function. This suggests that in the HTN groups the progression of the WM damage started earlier but also reached a plateau faster than in the corresponding CON groups.

Additionally, no significant hypertension effects, nor interactions were found for the FA measures in either of the structures. The hypertension-specific differences were observed solely in the remaining three coefficients (MD, RD, AD), suggesting an uneven sensitivity of the diffusion parameters for HTN-specific WM damage.

## Discussion

Our study tested for WM microstructure changes related to aging and hypertension. We explored spatial and functional patterns of DTI-derived diffusion coefficients’ deviations accompanying both factors, as well as possible independence/interdependence of their association with WM damage. Our results confirmed a destructive linkage, expressed by a decrease of the FA, and an increase in other diffusivity measures (MD, RD, AD) related to both hypertension and aging. At the same time – some variations were obvious. Brain asymmetry to hypertension-related WM degeneration was noted, where the greatest part of the hypertension specific effects were present in the left hemisphere. This spatial scheme was followed by a functional one. In most of the association tracts and the corpus callosum, an interdependence between aging and the HTN was noted, whereas in projection fibers and the fornix – the factors contributed independent of each other.

The hemispheres’ asymmetry in sensitivity to hypertension-related WM microstructure damage is probably the most intriguing of our results. As for aging, the differential pattern of the right and left hemisphere’s WM degeneration was neither clearly proven, nor negated yet, with some studies reporting significant differences (Zahr et al., 2009; Hasan et al., 2010; Michielse et al., 2010; Wahl et al., 2010; Lebel et al., 2012), and some reporting the lack of thereof (Stadlbauer et al., 2008; Lebel et al., 2012; Voineskos et al., 2012). It is remarkable considering certain theories that emphasize the role of the changes in the hemispheric asymmetry during aging. One of them is postulating a Hemispheric Asymmetry Reduction in OLD adults (HAROLD model), where during the lifespan the functional specialization of brain areas decreases, resulting in bilateral rather than lateralized processing of the information in the elderly population (Cabeza, 2002). The other is postulating greater age-related decline of the right hemisphere (the right-hemi model). Both theories have been grounded in several functional imaging studies (see Dolcos et al., 2002, for review), yet it is justified to speculate, that the mechanisms should be reflected in the WM structure as well. Our results shed some potential light on this discrepancy.

The pattern of effects associated with aging and hypertension which we found is hardly interpreted within the framework of the right-hemi theory. Admittedly, the large-scale analysis of the whole WM tissue degeneration showed age-related effects in the right hemisphere only, yet this pattern did not hold out against more precise measures (ones bound by specific tracts and diffusivity parameters). It could be speculated that the restriction of the hypertension-related changes to the left hemisphere’s WM may be the cause of the cognitive impairment often noted in the patients’ population (under the assumption that the normally slowly declining left hemisphere’s WM damage should have a noteworthy influence on a person’s cognitive outcome). Yet, according to this theory, the age-related effects ought to be more prominent in the right hemisphere irrespective of the level of specificity of the analyses (whether the whole tissue or particular pathways are considered), which is not the case. On the other hand, the HAROLD model provides a suitable context for the associations discovered. The model does not assume an asymmetry in hemispheres’ age-related deterioration, thus the ambiguous previous reports on the left to right alternations. However, in our study, we examined an additional noxious vascular factor and the way it adds up to the WM integrity measures decline. The left hemisphere’s WM vulnerability to hypertension that we found, may cause disruption, or decrease in the specificity of the neural transmission in the patient’s population. This would have led to a decrease in functional asymmetry – either due to compensation or dedifferentiation mechanisms as proposed by the model. This hypothesis is mildly supported by an fMRI study by our center showing a non-specific hypertension-related functional reorganization (Naumczyk et al., 2017).

Additionally, we found differential patterns of WM changes in tracts respective of their functional role. The immunity of the projection fibers to age-related deviations in diffusion parameters discovered by some of the previous reports (Stadlbauer et al., 2008; Voineskos et al., 2012) was scarcely confirmed by our results. Only MD, AD, and RD of the right and the AD of the left corticospinal tract transgressed the pattern of decline associated with age. Our results are closer to the ones described by Hasan et al. (2010) or Lebel et al. (2012), where aging-related changes were reflected in all compared fibers types and right hemisphere’s white matter. Yet, the functional classification of the pathways contributes to understanding the WM impairment associations of the second factor examined – hypertension. In projection fibers and in the fornix, if the disease-related effects were present, they occurred independent of the aging-related effects, whereas in the corpus callosum and most of the association fibers if hypertension contributed to diffusion parameters variance, an interaction with aging was observed. The only association fibers that did not follow this scheme were the right uncinate fasciculus and left cingulum in the cingulate cortex section. It should be also noted that the superior longitudinal fasciculus was the only tract out of examined ones, that did not present hypertension-related effects in either of the hemispheres.

The nature of the interaction observed is particularly interesting. The middle-aged group was the one mostly differentiating the HTN and CON, namely, these patients had a greater extent of WM microstructure damage than healthy peers. What is noteworthy is that this damage did not progress between the middle-aged and older patients, whereas in the controls – a decline was most prominent when comparing the aforementioned subgroups. In other words – the hypertensive patients’ association fibers of the left hemisphere tended to degenerate earlier than respective normotensive ones, and at the same time this impairment reached plateau faster in the HTN group resulting in no between-group differences in the older subjects. As many reports suggest a faster degeneration of WM of the association fibers (Bender and Raz, 2015; Bender et al., 2016), it may be speculated that this scheme reflects some acceleration in the left hemisphere’s WM aging associated with vascular degeneration.

The methodology used in our study (quantitative comparison of parameters derived from the whole brain and the tract volume), disallows a detailed reference to other spatial gradients reported previously – the anterior–posterior one and the superior-inferior one. Yet, it should be noted that we detected age-related effects in virtually all tracts when FA was compared. Also, most of the MD and the RD comparisons revealed sensitivity to aging and/or hypertension. The least sensitive parameter appeared to be the AD – in 10 out of 26 analyzed regions of interest no significant effects were found for either of the factors. This stays in line with some previous reports suggesting precedence of the RD coefficient (as related to AD) in pinpointing age-related WM damage (Davis et al., 2009; Lebel et al., 2012). Additionally, no hypertension related effects were present in FA comparisons of our study, only in other diffusivity measures (MD, AD, RD). This result is contradictory to previous studies (Kennedy and Raz, 2009; Maillard et al., 2012). This discrepancy may be due to differences in the hypertension group – in our study patients had well-controlled systolic and diastolic blood pressure. Despite the inconsistency mentioned, it should be emphasized that the WM integrity metrics proved sensitivity to hypertension-related brain damage (especially in the middle-aged group).

In this study, we showed that both age and hypertension are factors that influence white matter integrity, alas their interdependence varies among different fibers. Considering functional division, hypertension and age affected diffusion parameters in projection pathways as independent factors, whereas in association fibers an additional spatial gradient was observed. In the right hemisphere only age explained the diffusion decline in white matter of this hemisphere and almost all tracts (with an exception of the right uncinate fasciculus). In the left hemisphere the factors contribution to WM integrity disruption was intertwined. Hypertensive patients presented earlier aging increase in diffusion parameters, but after the rise in the middle-aged group, they reached a plateau with the same diffusion values as old healthy controls. This effect may be grounded in various mechanisms. It may be due to diastolic blood pressure that did not differ in the examined older subgroups, or maybe the WM’s integrity disruption rate was slowed by the intake of medications by the hypertensive patients. These suppositions should be verified in future longitudinal studies.

### Limitations

Our study has some limitations. First, BMI was not balanced in age groups between hypertension patients and healthy controls. Despite this difference in our study, there were no reasonable correlations between the BMI scores and dependent variables of interest (the diffusion parameters) in the analyzed data, thus not supporting the rationale of including one’s weight as an additional covariate. Furthermore, all of the mean BMIs were within the range of an overweight rank in NIH scale.

Additionally, we did not explore the effects of the hypertension duration on results obtained, which in some studies proved a significant factor (Mcevoy et al., 2015). Yet, usually determining the actual hypertension onset is not fully reliable, as the disease may progress without noticeable symptoms.

Furthermore, we did not mask out the WMHs occurring in the subjects’ white matter tissue. The WMHs can affect the diffusion coefficients of a given region of interest and thus alter the group comparisons. Still, the HTN and CON subjects did not differ in terms of WMH burden (as assessed by the Fazekas scale), it rather gradually increased with age, irrespective of the disease status. Also, the Fazekas scores scarcely exceeded what is considered the low level WMHs (the scale’s scores of 0 and 1 dominated in the group). On top of that, there was no specific pattern of the WMHs topography – in other words the pathways were affected randomly not recurrently and thus these occurrences should not influence the major conclusions drawn about the intergroup differences.

Additionally, our study was a cross-sectional one, which disallows evaluation of the causation of the variables. Further longitudinal studies are needed to investigate the mechanism underlying the relationships between hypertension, age and the white matter integrity we observed. Finally, the medication intake in the patients group might have also influenced the results.

## Author Contributions

AS, AG, PN, AJ, and AK: acquisition. AS, PN, and AM contributed to the analysis or the interpretation of data. PN, AS, and BG drafted the manuscript. ES, KN, KJ, DG, MW, and JK critically revised the manuscript for important intellectual content.

## Conflict of Interest Statement

The authors declare that the research was conducted in the absence of any commercial or financial relationships that could be construed as a potential conflict of interest.

## References

[B1] Allen B. Muldoon M. F. Gianaros P. J. Jennings J. R. (2016). Higher blood pressure partially links greater adiposity to reduced brain white matter integrity. *Am. J. Hypertens.* 29 1029–1037. 10.1093/ajh/hpw026 26970287 PMC5661494

[B2] Behrens T. E. J. Woolrich M. W. Jenkinson M. Johansen-Berg H. Nunes R. G. Clare S. (2003). Characterization and propagation of uncertainty in diffusion-weighted MR imaging. *Magn. Reson. Med.* 50 1077–1088. 10.1002/mrm.10609 14587019

[B3] Bender A. R. Raz N. (2015). Normal-appearing cerebral white matter in healthy adults: mean change over 2 years and individual differences in change. *Neurobiol. Aging* 36 1834–1848. 10.1016/j.neurobiolaging.2015.02.001 25771392 PMC4419147

[B4] Bender A. R. Völkle M. C. Raz N. (2016). Differential aging of cerebral white matter in middle-aged and older adults: a seven-year follow-up. *Neuroimage* 125 74–83. 10.1016/j.neuroimage.2015.10.030 26481675 PMC4691398

[B5] Bennett I. J. Greenia D. E. Maillard P. Sajjadi S. A. DeCarli C. Corrada M. M. (2017). Age-related white matter integrity differences in oldest-old without dementia. *Neurobiol. Aging* 56 108–114. 10.1016/j.neurobiolaging.2017.04.013 28527525 PMC5647141

[B6] Bennett I. J. Madden D. J. Vaidya C. J. Howard D. V. Howard J. H. (2010). Age-related differences in multiple measures of white matter integrity: a diffusion tensor imaging study of healthy aging. *Hum. Brain Mapp.* 31 378–390. 10.1002/hbm.20872 19662658 PMC2826569

[B7] Bucur B. Madden D. J. Spaniol J. Provenzale J. M. Cabeza R. White L. E. (2008). Age-related slowing of memory retrieval: contributions of perceptual speed and cerebral white matter integrity. *Neurobiol. Aging* 29 1070–1079. 10.1016/j.neurobiolaging.2007.02.008 17383774 PMC2396513

[B8] Cabeza R. (2002). Hemispheric asymmetry reduction in older adults: the harold model. *Psychol. Aging* 17 85–100. 10.1037/0882-7974.17.1.85 11931290

[B9] Chen X. Wen W. Anstey K. J. Sachdev P. S. (2006). Effects of cerebrovascular risk factors on gray matter volume in adults aged 60–64 years: a voxel-based morphometric study. *Psychiatry Res. Neuroimaging* 147 105–114. 10.1016/j.pscychresns.2006.01.009 16962291

[B10] Davis S. W. Dennis N. A. Buchler N. G. White L. E. Madden D. J. Cabeza R. (2009). Assessing the effects of age on long white matter tracts using diffusion tensor tractography. *Neuroimage* 46 530–541. 10.1016/j.neuroimage.2009.01.068 19385018 PMC2775533

[B11] de Groot M. Cremers L. G. M. Ikram M. A. Hofman A. Krestin G. P. van der Lugt A. (2016). White matter degeneration with aging: longitudinal diffusion MR imaging analysis. *Radiology* 279 532–541. 10.1148/radiol.2015150103 26536311

[B12] den Heijer T. Launer L. J. Prins N. D. van Dijk E. J. Vermeer S. E. Hofman A. (2005). Association between blood pressure, white matter lesions, and atrophy of the medial temporal lobe. *Neurology* 64 263–267. 10.1212/01.WNL.0000149641.55751.2E 15668423

[B13] Dickie D. A. Ritchie S. J. Cox S. R. Sakka E. Royle N. A. Aribisala B. S. (2016). Vascular risk factors and progression of white matter hyperintensities in the Lothian Birth Cohort 1936. *Neurobiol. Aging* 42 116–123. 10.1016/j.neurobiolaging.2016.03.011 27143428 PMC4869590

[B14] Dolcos F. Rice H. J. Cabeza R. (2002). Hemispheric asymmetry and aging: right hemisphere decline or asymmetry reduction. *Neurosci. Biobehav. Rev.* 26 819–825. 10.1016/S0149-7634(02)00068-4 12470693

[B15] Fazekas F. Chawluk J. Alavi A. Hurtig H. Zimmerman R. (1987). MR signal abnormalities at 1.5 T in Alzheimer’s dementia and normal aging. *Am. J. Roentgenol.* 149 351–356. 10.2214/ajr.149.2.351 3496763

[B16] Fazekas F. Kleinert R. Offenbacher H. Schmidt R. Kleinert G. Payer F. (1993). Pathologic correlates of incidental MRI white matter signal hyperintensities. *Neurology* 43 1683–1689. 10.1212/WNL.43.9.1683 8414012

[B17] Gons R. A. R. Van Oudheusden L. J. B. De Laat K. F. Van Norden A. G. W. Van Uden I. W. M. Norris D. G. (2012). Hypertension is related to the microstructure of the corpus callosum: the RUN DMC study. *J. Alzheimer’s Dis.* 32 623–631. 10.3233/JAD-2012-121006 22869466

[B18] Hasan K. M. Kamali A. Abid H. Kramer L. A. Fletcher J. M. Ewing-Cobbs L. (2010). Quantification of the spatiotemporal microstructural organization of the human brain association, projection and commissural pathways across the lifespan using diffusion tensor tractography. *Brain Struct. Funct.* 214 361–373. 10.1007/s00429-009-0238-0 20127357 PMC2864323

[B19] Jenkinson M. Beckmann C. F. Behrens T. E. J. Woolrich M. W. Smith S. M. (2012). FSL. *Neuroimage* 62 782–790. 10.1016/J.NEUROIMAGE.2011.09.015 21979382

[B20] Jones D. K. (2008). Studying connections in the living human brain with diffusion MRI. *Cortex* 44 936–952. 10.1016/j.cortex.2008.05.002 18635164

[B21] Kennedy K. M. Raz N. (2009). Pattern of normal age-related regional differences in white matter microstructure is modified by vascular risk. *Brain Res.* 1297 41–56. 10.1016/j.brainres.2009.08.058 19712671 PMC2758325

[B22] Lebel C. Gee M. Camicioli R. Wieler M. Martin W. Beaulieu C. (2012). Diffusion tensor imaging of white matter tract evolution over the lifespan. *Neuroimage* 60 340–352. 10.1016/j.neuroimage.2011.11.094 22178809

[B23] Leemans A. Jeurissen B. Sijbers J. Jones D. (2009). ExploreDTI: a graphical toolbox for processing, analyzing, and visualizing diffusion MR data. *Proc. Int. Soc. Magn. Reson. Med.* 17:3537.

[B24] Leemans A. Jones D. K. (2009). The B-matrix must be rotated when correcting for subject motion in DTI data. *Magn. Reson. Med.* 61 1336–1349. 10.1002/mrm.21890 19319973

[B25] Maillard P. Seshadri S. Beiser A. Himali J. J. Au R. Fletcher E. (2012). Effects of systolic blood pressure on white-matter integrity in young adults in the framingham heart study: a cross-sectional study. *Lancet Neurol.* 11 1039–1047. 10.1016/S1474-4422(12)70241-7 23122892 PMC3510663

[B26] Mårtensson J. Lätt J. Åhs F. Fredrikson M. Söderlund H. Schiöth H. B. (2017). Diffusion tensor imaging and tractography of the white matter in normal aging: the rate-of-change differs between segments within tracts. *Magn. Reson. Imaging* 45 113–119. 10.1016/j.mri.2017.03.007 28359912

[B27] Mcevoy L. K. Fennema-Notestine C. Eyler L. T. Franz C. E. Hagler D. J. Lyons M. J. (2015). Hypertension-related alterations in white matter microstructure detectable in middle age. *Hypertension* 66 317–323. 10.1161/HYPERTENSIONAHA.115.05336 26056337 PMC4499000

[B28] Michielse S. Coupland N. Camicioli R. Carter R. Seres P. Sabino J. (2010). Selective effects of aging on brain white matter microstructure: a diffusion tensor imaging tractography study. *Neuroimage* 52 1190–1201. 10.1016/j.neuroimage.2010.05.019 20483378

[B29] Naumczyk P. Sabisz A. Witkowska M. Graff B. Jodzio K. Gąsecki D. (2017). Compensatory functional reorganization may precede hypertension-related brain damage and cognitive decline: a functional magnetic resonance imaging study. *J. Hypertens.* 35 1252–1262. 10.1097/HJH.0000000000001293 28169883 PMC5404398

[B30] Oishi K. Zilles K. Amunts K. Faria A. Jiang H. Li X. (2008). Human brain white matter atlas: identification and assignment of common anatomical structures in superficial white matter. *Neuroimage* 43 447–457. 10.1016/j.neuroimage.2008.07.009 18692144 PMC2586008

[B31] Raz N. Lindenberger U. Rodrigue K. M. Kennedy K. M. Head D. Williamson A. (2005). Regional brain changes in aging healthy adults: general trends, individual differences and modifiers. *Cereb. Cortex* 15 1676–1689. 10.1093/cercor/bhi044 15703252

[B32] Raz N. Rodrigue K. M. Haacke E. M. (2007a). Brain aging and its modifiers: insights from in vivo neuromorphometry and susceptibility weighted imaging. *Ann. N. Y. Acad. Sci.* 1097 84–93. 10.1196/annals.1379.018 17413014 PMC2630248

[B33] Raz N. Rodrigue K. M. Kennedy K. M. Acker J. D. (2007b). Vascular health and longitudinal changes in brain and cognition in middle-aged and older adults. *Neuropsychology* 21 149–157. 10.1037/0894-4105.21.2.149 17402815

[B34] Salat D. H. Tuch D. S. Greve D. N. van der Kouwe A. J. W. Hevelone N. D. Zaleta A. K. (2005). Age-related alterations in white matter microstructure measured by diffusion tensor imaging. *Neurobiol. Aging* 26 1215–1227. 10.1016/j.neurobiolaging.2004.09.017 15917106

[B35] Scott J. A. Braskie M. N. Tosun D. Thompson P. M. Weiner M. DeCarli C. (2015). Cerebral amyloid and hypertension are independently associated with white matter lesions in elderly. *Front. Aging Neurosci.* 7:221 10.3389/fnagi.2015.00221 PMC4664630 26648866

[B36] Shrestha I. Takahashi T. Nomura E. Ohtsuki T. Ohshita T. Ueno H. (2009). Association between central systolic blood pressure, white matter lesions in cerebral MRI and carotid atherosclerosis. *Hypertens. Res.* 32 869–874. 10.1038/hr.2009.121 19644503

[B37] Song S. K. Sun S. W. Ju W. K. Lin S. J. Cross A. H. Neufeld A. H. (2003). Diffusion tensor imaging detects and differentiates axon and myelin degeneration in mouse optic nerve after retinal ischemia. *Neuroimage* 20 1714–1722. 10.1016/j.neuroimage.2003.07.005 14642481

[B38] Song S. K. Yoshino J. Le T. Q. Lin S. J. Sun S. W. Cross A. H. (2005). Demyelination increases radial diffusivity in corpus callosum of mouse brain. *Neuroimage* 26 132–140. 10.1016/j.neuroimage.2005.01.028 15862213

[B39] Stadlbauer A. Salomonowitz E. Strunk G. Hammen T. Ganslandt O. (2008). Age-related degradation in the central nervous system: assessment with diffusion-tensor imaging and quantitative fiber tracking. *Radiology* 247 179–188. 10.1148/radiol.2471070707 18292477

[B40] Stanek K. M. Grieve S. M. Brickman A. M. Korgaonkar M. S. Paul R. H. Cohen R. A. (2011). Obesity is associated with reduced white matter integrity in otherwise healthy adults^∗^. *Obesity* 19 500–504. 10.1038/oby.2010.312 21183934

[B41] Strassburger T. L. Lee H. C. Daly E. M. Szczepanik J. Krasuski J. S. Mentis M. J. (1997). Interactive effects of age and hypertension on volumes of brain structures. *Stroke* 28 1410–1417. 10.1161/01.STR.28.7.1410 9227693

[B42] Sullivan E. V. Adalsteinsson E. Hedehus M. Ju C. Moseley M. Lim K. O. (2001). Equivalent disruption of regional white matter microstructure in ageing healthy men and women. *Neuroreport* 12 99–104. 10.1097/00001756-200101220-00027 11201100

[B43] Sullivan E. V. Rohlfing T. Pfefferbaum A. (2010a). Longitudinal study of callosal microstructure in the normal adult aging brain using quantitative DTI fiber tracking. *Dev. Neuropsychol.* 35 233–256. 10.1080/87565641003689556 20446131 PMC2867078

[B44] Sullivan E. V. Rohlfing T. Pfefferbaum A. (2010b). Quantitative fiber tracking of lateral and interhemispheric white matter systems in normal aging: relations to timed performance. *Neurobiol. Aging* 31 464–481. 10.1016/j.neurobiolaging.2008.04.007 18495300 PMC2815144

[B45] Sullivan E. V. Zahr N. M. Rohlfing T. Pfefferbaum A. (2010c). Fiber tracking functionally distinct components of the internal capsule. *Neuropsychologia* 48 4155–4163. 10.1016/j.neuropsychologia.2010.10.023 20974161 PMC2993875

[B46] Taki Y. Goto R. Evans A. Zijdenbos A. Neelin P. Lerch J. (2004). Voxel-based morphometry of human brain with age and cerebrovascular risk factors. *Neurobiol. Aging* 25 455–463. 10.1016/j.neurobiolaging.2003.09.002 15013566

[B47] Tournier J.-D. Mori S. Leemans A. (2011). Diffusion tensor imaging and beyond. *Magn. Reson. Med.* 65 1532–1556. 10.1002/mrm.22924 21469191 PMC3366862

[B48] Voineskos A. N. Rajji T. K. Lobaugh N. J. Miranda D. Shenton M. E. Kennedy J. L. (2012). Age-related decline in white matter tract integrity and cognitive performance: a DTI tractography and structural equation modeling study. *Neurobiol. Aging* 33 21–34. 10.1016/j.neurobiolaging.2010.02.009 20363050 PMC2945445

[B49] Wahl M. Li Y. O. Ng J. LaHue S. C. Cooper S. R. Sherr E. H. (2010). Microstructural correlations of white matter tracts in the human brain. *Neuroimage* 51 531–541. 10.1016/j.neuroimage.2010.02.072 20206699 PMC2856800

[B50] Winklewski P. J. Sabisz A. Naumczyk P. Jodzio K. Szurowska E. Szarmach A. (2018). Understanding the physiopathology behind axial and radial diffusivity changes-what do we know? *Front. Neurol.* 9:92. 10.3389/fneur.2018.00092 29535676 PMC5835085

[B51] Xu J. Li Y. Lin H. Sinha R. Potenza M. N. (2013). Body mass index correlates negatively with white matter integrity in the fornix and corpus callosum: a diffusion tensor imaging study. *Hum. Brain Mapp.* 34 1044–1052. 10.1002/hbm.21491 22139809 PMC3314715

[B52] Zahr N. M. Rohlfing T. Pfefferbaum A. Sullivan E. V. (2009). Problem solving, working memory, and motor correlates of association and commissural fiber bundles in normal aging: a quantitative fiber tracking study. *Neuroimage* 44 1050–1062. 10.1016/j.neuroimage.2008.09.046 18977450 PMC2632960

