# Supplementary material for: Aging and Hypertension – Independent or Intertwined White Matter Impairing Factors? Insights From the Quantitative Diffusion Tensor Imaging
Source: Front Aging Neurosci. 2019 Feb 19;11:35. doi: 10.3389/fnagi.2019.00035 (PMC6389787; doi:10.3389/fnagi.2019.00035)
Supplement: Supplementary file 1 [file Data_Sheet_1.PDF]

Supplementary Material

The Pearson's and Spearman's correlation coefficients for all the diffusivity measures and the subjects weight parameters are presented in the Table SM1 below. None of the correlations reached reasonable value (absolute value of over 0.3), therefore these variables were not included as covariates in the final model.

Signatures of the tracts are as follows (L – left, R – right):

L1 – Anterior thalamic radiation L, R1 – Anterior thalamic radiation R,
L2 – Corticospinal tract L, R2 – Corticospinal tract R
L3 – Cingulum (hippocampus) L, R3 – Cingulum (hippocampus)
L4 – Cingulum (cingulate gyrus) L, R4 – Cingulum (cingulate gyrus) R
L5 – Inferior fronto-occipital fasciculus L, R5 – Inferior fronto-occipital fasciculus R
L6 – Inferior longitudinal fasciculus L, R6 – Inferior longitudinal fasciculus R
L7 – Superior longitudinal fasciculus L, R7 – Superior longitudinal fasciculus R
L8 – Superior longitudinal fasciculus (temporal part) L, R8 – Superior longitudinal fasciculus (temporal part) R
L9 – Uncinate fasciculus L, R9 – Uncinate fasciculus R
CC – Corpus callosum, FX - Fornix.
LH – left hemisphere, RH – right hemisphere
WM – white matter

Table SM1. The Pearson's and Spearmans's correlation coefficients for the body mass index (BMI) and the waist-to-hip ratio (WHR) and all of the diffusivity parameters.

	FA		MD		RD		AD	
	WHR	BMI	WHR	BMI	WHR	BMI	WHR	BMI
brain	0.014	-0.094	0.089	0.052	0.081	0.059	0.105	0.038
wm	0.034	-0.092	0.055	0.057	0.039	0.070	0.083	0.025
CC	-0.116	-0.016	0.164	0.062	0.163	0.068	0.157	0.047
FX	-0.089	-0.056	0.216	0.190	0.201	0.181	0.228	0.197
L1	-0.042	-0.009	0.229	0.208	0.223	0.198	0.247	0.240
L2	-0.112	0.004	0.154	0.093	0.173	0.092	0.086	0.075
L3	0.000	0.039	0.133	0.088	0.133	0.105	0.106	0.056
L4	-0.015	0.097	0.080	-0.022	0.063	-0.049	0.088	0.029
L5	-0.113	0.020	0.156	0.003	0.154	0.012	0.147	-0.018
L6	-0.122	0.060	0.135	-0.023	0.139	-0.019	0.111	-0.039
L7	-0.189	0.030	0.230	0.090	0.242	0.093	0.197	0.084
L8	-0.204	0.001	0.191	0.024	0.217	0.034	0.116	-0.013
L9	-0.058	0.089	0.148	0.047	0.147	0.029	0.126	0.076
left_hemi_brain	0.014	-0.106	0.116	0.061	0.104	0.066	0.137	0.051
left_hemi_wm	0.035	-0.107	0.081	0.092	0.058	0.099	0.123	0.072
R1	-0.031	0.002	0.246	0.221	0.237	0.220	0.267	0.239
R2	-0.082	0.058	0.108	0.020	0.137	0.028	0.038	0.001
R3	0.089	0.153	0.108	-0.017	0.093	-0.006	0.115	-0.047
R4	-0.087	0.053	0.046	-0.089	0.075	-0.074	-0.004	-0.094
R5	-0.096	0.081	0.079	-0.074	0.089	-0.064	0.047	-0.087
R6	-0.130	0.094	0.061	-0.162	0.078	-0.144	0.023	-0.175
R7	-0.188	0.042	0.171	0.030	0.194	0.038	0.121	0.014
R8	-0.184	0.075	0.133	-0.010	0.164	-0.007	0.068	-0.014
R9	-0.040	0.150	0.128	0.052	0.125	0.036	0.129	0.081
right_hemi_brain	0.014	-0.081	0.061	0.042	0.056	0.050	0.070	0.024
right_hemi_wm	0.032	-0.075	0.030	0.023	0.020	0.041	0.044	-0.015

The detailed results of two-way ANOVA of age and hypertension on diffusion parameters in the whole brain, the whole white matter, the left hemisphere and the right hemisphere and white matter of each of the hemisphere's and also twenty tracts are presented below, divided by diffusion coefficients. The means and standard deviations of diffusion coefficient in different white matter tissues are presented on charts (SM_Figure_all). If the interaction between hypertension and age appeared significant, the T-test based contrasts were analyzed to check the simple effects.

a) Fractional anisotropy FA

The two-way ANOVA of FA for each tract showed:

- No interaction between age and hypertension factors;
- A significant main effect of age was found in almost all tracts. FA coefficient was decreasing with age. The difference was found in following tracts: L1 ($F=9.874$, $p<0.001$), R1 ($F=7.665$, $p=0.001$), CC ($F=9.419$, $p<0.001$), L2 ($F=12.000$ $p<0.001$), R2 ($F=6.545$, $p=0.002$), FX ($F=11.570$, $p<0.001$), L3 ($F=6.440$, $p=0.002$), L4 ($F=5.315$, $p=0.006$), R4 ($F=5.014$, $p=0.008$), L5 ($F=11.663$, $p<0.001$), R5 ($F=8.185$, $p<0.001$), L6 ($F=13.610$, $p<0.001$), R6 ($F=9.392$, $p<0.001$), L7 ($F=9.486$, $p<0.001$), R7 ($F=7.074$, $p=0.001$), L8 ($F=7.978$, $p=0.001$), R8 ($F=6.875$, $p=0.001$), L9 ($F=4.706$, $p=0.011$);
- No significant main effect for hypertension factor was found.

No effect of age and disease was found in whole brain, whole brain WM, LH brain, LH white matter, RH brain, RH white matter, right cingulum (hippocampal part) - R3 and right uncinate fasciculus - R9.

b) Mean diffusivity MD

The two-way ANOVA of MD for each tract showed:

- Significant interaction between age and hypertension factors in CC ($F=4.560$, $p=0.012$), L3 ($F=3.219$, $p=0,043$), L5 ($F=3.638$, $p=0.029$), L6 ($F=4.257$, $p=0.016$), L9 ($F=3.467$, $p=0.034$);
- A significant main effect of age was found in several tracts. MD coefficient was increasing with age. The difference was found in following tracts: whole brain WM ($F=3.309$, $p=0.039$), RH white matter ($F=3.732$, $p=0.026$), L1 ($F=9.362$, $p<0.001$), R1 ($F=11.758$, $p<0.001$), L2 ($F=6.546$, $p=0.002$), FX ($F=12.230$, $p<0.001$), R5 ($F=4.949$, $p=0.008$), L7 ($F=20.317$, $p<0.001$), R7 ($F=5.748$, $p=0.004$), L8 ($F=13.753$, $p<0.001$), R8 ($F=4.334$, $p=0.015$), R9 ($F=10.030$, $p<0.001$);
- A main effect for hypertension factor was found in L1 ($F=4.732$, $p=0.031$), R2 ($F=5.285$, $p=0.023$), FX ($F=4.315$, $p=0.040$), L4 ($F=4.297$, $p=0.040$), R9 ($F=4,892$, $p=0.029$). The HTN subgroup had a higher MD coefficient than the CON subgroup in this projections.

No effect of age and disease was found in the whole brain, LH brain, LH white matter, RH brain, right cingulum cingulate gyrus – R4, right cingulum (hippocampal part) - R3 and right inferior longitudinal fasciculus - R6.

Corpus callosum showed a significant interaction, a simple effects t-test analyses were performed to investigate the nature of the interaction. There was a significant effect between control and hypertension group in age group II ($p<0.001$). These hypertension patients had higher MD coefficient than healthy controls. In control group, there was a significant effect between age group I and III ($p<0.001$) and group II and III ($p=0.002$). In the disease group, there was a significant effect between group I and II ($p=0.042$). In each difference older participants had higher MD coefficient.

Left cingulum (hippocampal part) (L3) presented significant interactions. There was a significant effect in control group between age group II and III (p=0.003), in hypertension group an effect between age group I and III (p<0.001). Older individuals had higher values of mean diffusivity.

Simple effects were calculated in left inferior fronto-occipital fasciculus (L5). A significant effect between control and disease group in age group II (p=0.012) was shown. Hypertension patients had higher MD coefficient than controls. There was a significant effect in control group between age group I and III (p<0.001) group II and III (p=0.001), in HTN group an effect between age group I and II (p=0.005) and in the age group I and III (p=0.001). In each difference mean diffusivity increased with age.

Left inferior longitudinal fasciculus (L6) showed a significant interaction, a simple effects analyses were performed to investigate the nature of the interaction. There was a significant effect between control and hypertension group in age group II (p=0.049). Patients had higher MD coefficient than controls. In control group, there was a significant effect between age group I and III (p<0.001) and group II and III (p<0.001). In HTN group, there was a significant effect between group I and II (p=0.004) and in the age group I and III (p<0.001). In each difference older members had higher values of MD coefficient.

Simple effects were calculated in left uncinate fasciculus (L9) and results presented: a significant effect between control and subject group in age group II (p=0.003), in control group an effect between age group I and III (p<0.001) and in age group II and III (p=0.003), in disease group an effect between age group I and II (p=0.011) and in age group I and III (p=0.008). The relationship between age and hypertension in values of mean diffusivity is the same as in other tracts which had interaction effect. Subjects had higher MD coefficient than controls in age group II and older participants had higher values of mean diffusivity.

c) Radial diffusivity RD

The two-way ANOVA of RD for each tract showed:

- Significant interaction between age and hypertension factors in CC (F=4.430, p=0.014), L3 (F=3.505, p=0.033), L5 (F=3.328, p=0.039), L6 (F=3.831, p=0.024), L9 (F=3.237, p=0.042);
- A significant main effect of age was found in several tracts. RD coefficient was increasing with age. The difference was found in following tracts: whole brain white matter (F=3.350, p=0.038), RH white matter (F=3.751, p=0.026), L1 (F=10.241, p<0.001), R1 (F=11.463, p<0.001), L2 (F=11.245, p<0.001), FX (F=12.718, p<0.001), R3 (F=3.743, p=0.026), L4 (F=3.444, p=0.035), R5 (F=7.311, p=0.001), R6 (F=6.023, p=0.003), L7 (F=20.306, p<0.001), R7 (F=7.282, p=0.001), L8 (F=14.035, p<0.001), R8 (F=6.030, p=0.003), R9 (F=9.573, p<0.001);
- A main effect for hypertension factor was found in L1 (F=4.029, p=0.047), R9 (F=3.894, p=0.050). The HTN subgroup had a higher RD coefficient than the CON subgroup in this projections.

No effect of age and disease was found in the whole brain, LH brain, LH white matter, RH brain, right cingulum cingulate gyrus – R4 and right corticospinal tract – R2.

Corpus callosum showed a significant interaction, a simple effects analyses were performed to investigate the nature of the interaction. There was a significant effect between control and

hypertension group in age group II (p<0.001). Subjects had higher RD coefficient than controls. In control group, there was a significant effect between age group I and III (p<0.001) and group II and III (p=0.002). In disease group, there was a significant effect between group I and II (p=0.013) and the group I and III (p=0.033). Older participants had higher values of radial diffusivity.

Left cingulum (hippocampal part) (L3) presented interaction. In control group there was an effect between age group II and III (p=0.001), in hypertension group effects was shown between age group I and II (p=0.041) and in the age group I and III (p<0.001). Older individuals had higher values of radial diffusivity.

Simple effects were calculated in left inferior fronto-occipital fasciculus (L5) and results presented: a significant effect between control and subject group in age group II (p=0.011). Patients with hypertension had higher RD coefficient. There were effects: in controls between age group I and III (p<0.001), group II and III (p=0.002), in disease group an effect between age group I and II (p=0.001) and in the age group I and III (p<0.001). The same as earlier tracts, older participants had higher values of RD.

Left inferior longitudinal fasciculus (L6) showed a significant interaction. In control group, there was a significant effect between age group I and III (p<0.001) and group II and III (p<0.001). In the hypertension group, there was a significant effect between group I and II (p=0.001) and in the age group I and III (p<0.001). Older individuals had higher values of radial diffusivity.

Simple effects were calculated in left uncinate fasciculus (L9) and results showed: a significant effect between control and HTN group in age group II (p=0.004), in control group an effect between age group I and III (p=0.001), group II and III (p=0.007), in disease group an effect between age group I and II (p=0.005) and in age group I and III (p=0.004). The relationship between age and hypertension in values of radial diffusivity in L9 tract is similar to further tracts which had interaction effect. Subjects had higher RD coefficient than controls in age group II and older participants had higher values of radial diffusivity.

d) Axial diffusivity AD

The two-way ANOVA of AD for each tract showed:

- Significant interaction between age and hypertension factors in CC (F=4.355, p=0.015), L6 (F=3.677, p=0.028), L9 (F=4.371, p=0.014);
- A significant main effect of age was found in several tracts. AD coefficient was increasing with age. The difference was found in following tracts: RH white matter (F=3.319, p=0.039), L1 (F=7.800, p=0.001), R1 (F=9.544, p<0.001), L3 (F=8.718, p=0<0.001), FX (F=11.176, p<0.001), L5 (F=7.924, p=0.001), L7 (F=16.440, p<0.001), R7 (F=3.273, p=0.041), L8 (F=8.586, p<0.001), R9 (F=9.764, p<0.001),
- A significant main effect of hypertension was found in several tracts. The HTN subgroup had a higher AD coefficient than the CON subgroup. The difference was found in following tracts: L1 (F=5.795, p=0.017), L2 (F=4.331, p=0.039), R2 (F=7.863, p=0.006), FX (F=5.226, p=0.024), L4 (F=4.157, p=0,043), R9 (F=6.735, p=0.010).

No effect of age and disease was found in the whole brain, whole brain WM, LH brain, LH white matter, RH brain, right cingulum cingulate gyrus – R4, right cingulum (hippocampal part) - R3,

right inferior fronto-occipital fasciculus - R5, right inferior longitudinal fasciculus - R6, right superior longitudinal fasciculus (temporal part) - R8.

Corpus callosum presented significant interactions. Simple effects showed: an effect between control and hypertension group in the age group I ($p=0.027$) and also in age group II ($p=0.001$), in control group there was a significant effect between age group I and III ($p=0.002$), group II and III ($p=0.007$).

Left inferior longitudinal fasciculus (L6) showed a significant interaction, a simple effects analyses were performed to investigate the nature of the interaction. In control group, there was a significant effect between age group I and III ($p=0.009$) and group II and III ($p<0.001$). In HTN group, there was a significant effect between age group I and III ($p=0.001$). Older individuals had higher values of radial diffusivity.

Simple effects were calculated in left uncinate fasciculus (L9) and presented: a significant effect between control and subject group in age group II ($p=0.004$). Patients with hypertension had higher AD coefficient. In control group, there was a significant effect between age group I and III ($p<0.001$), group II and III ($p<0.001$). In each difference, axial diffusivity increased with age.
