# Supplementary material for: Aging and Hypertension – Independent or Intertwined White Matter Impairing Factors? Insights From the Quantitative Diffusion Tensor Imaging
Source: Front Aging Neurosci. 2019 Feb 19;11:35. doi: 10.3389/fnagi.2019.00035 (PMC6389787; doi:10.3389/fnagi.2019.00035)
Supplement: Supplementary file 2 [file Data_Sheet_2.PDF]

Whole brain

Fractional anisotropy

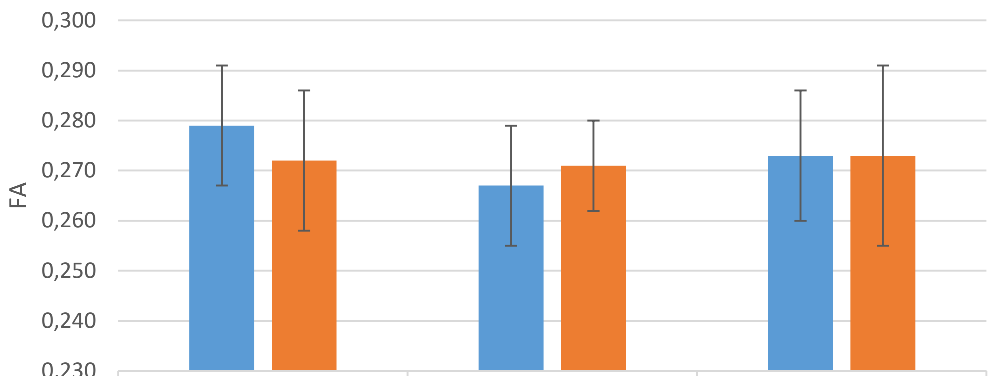

	1	2	3
CON	0,279	0,267	0,273
HTN	0,272	0,271	0,273

Radial diffusivity

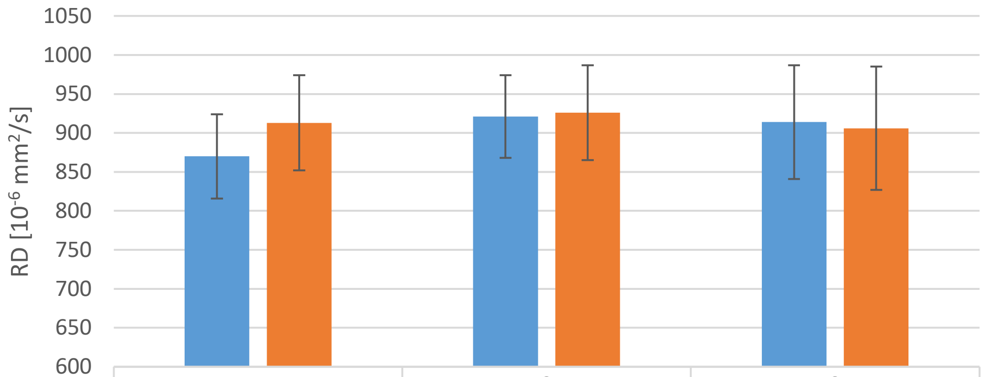

	1	2	3
CON	870	921	914
HTN	913	926	906

Mean diffusivity

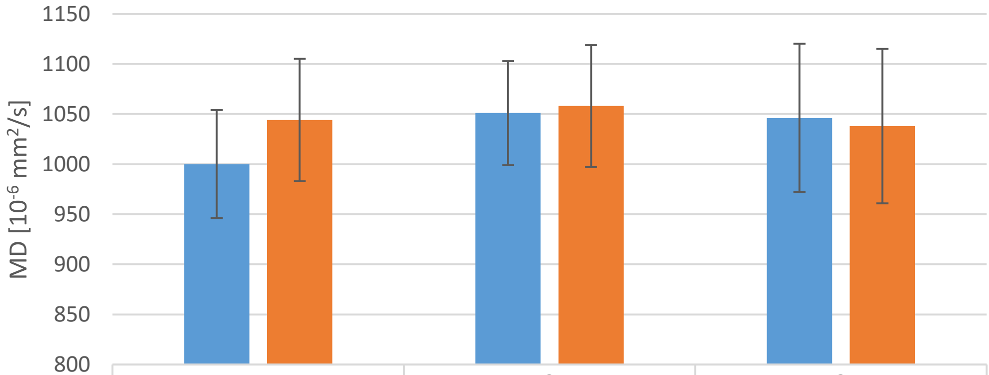

	1	2	3
CON	1000	1051	1046
HTN	1044	1058	1038

Axial diffusivity

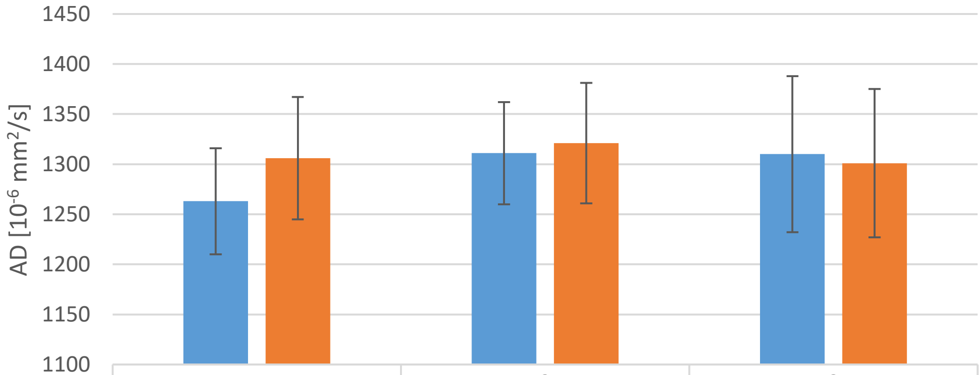

	1	2	3
CON	1263	1311	1310
HTN	1306	1321	1301

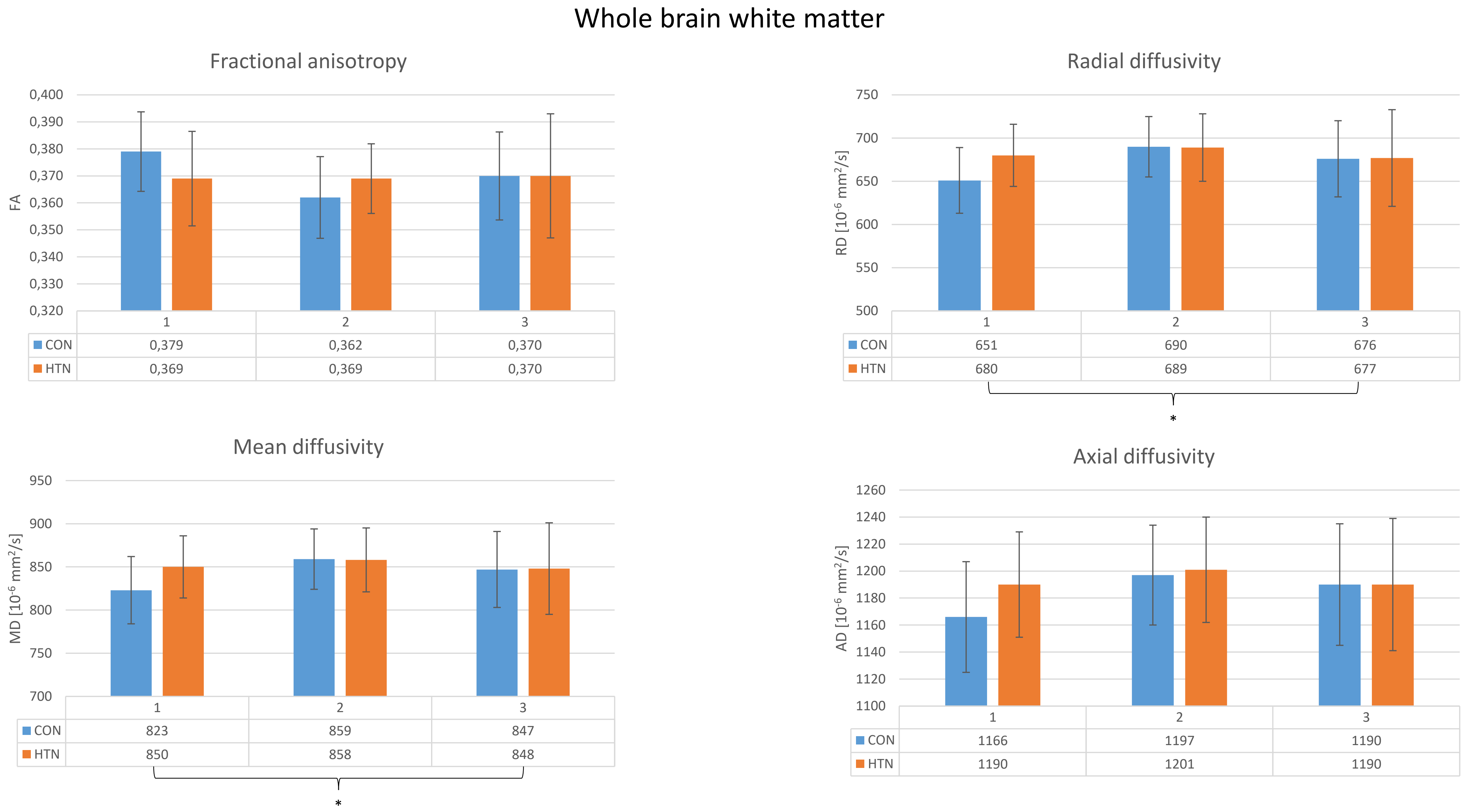
Whole brain white matter
Fractional anisotropy
FA
0,400
0,390
0,380
0,370
0,360
0,350
0,340
0,330
0,320
1
2
3
CON 0,379 0,362 0,370
HTN 0,369 0,369 0,370
Radial diffusivity
RD [10-6 mm2/s]
750
700
650
600
550
500
1
2
3
CON 651 690 676
HTN 680 689 677
*
Mean diffusivity
MD [10-6 mm2/s]
950
900
850
800
750
700
1
2
3
CON 823 859 847
HTN 850 858 848
*
Axial diffusivity
AD [10-6 mm2/s]
1260
1240
1220
1200
1180
1160
1140
1120
1100
1
2
3
CON 1166 1197 1190
HTN 1190 1201 1190

Left hemisphere brain

Fractional anisotropy

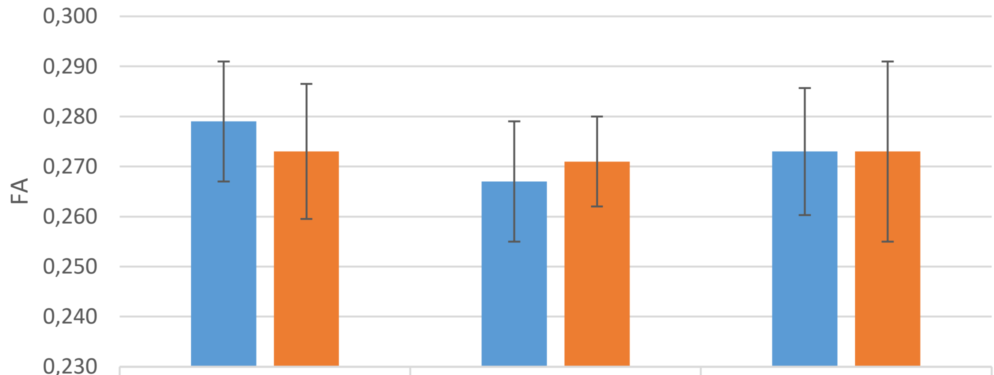

	1	2	3
CON	0,279	0,267	0,273
HTN	0,273	0,271	0,273

Radial diffusivity

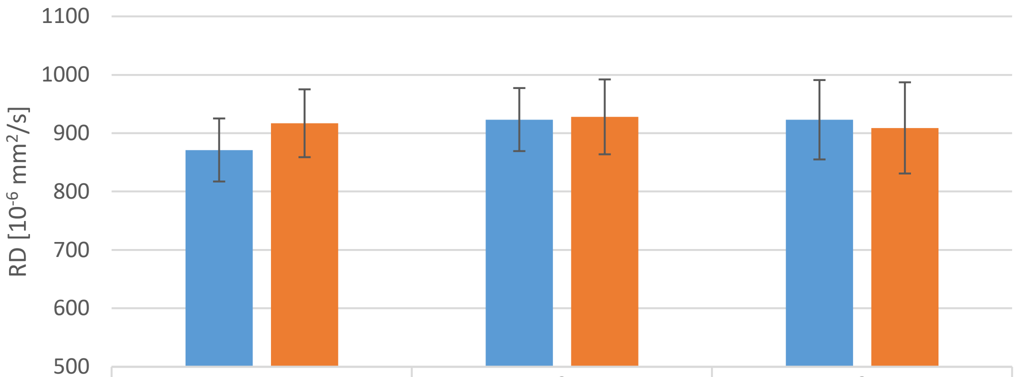

	1	2	3
CON	871	923	923
HTN	917	928	909

Mean diffusivity

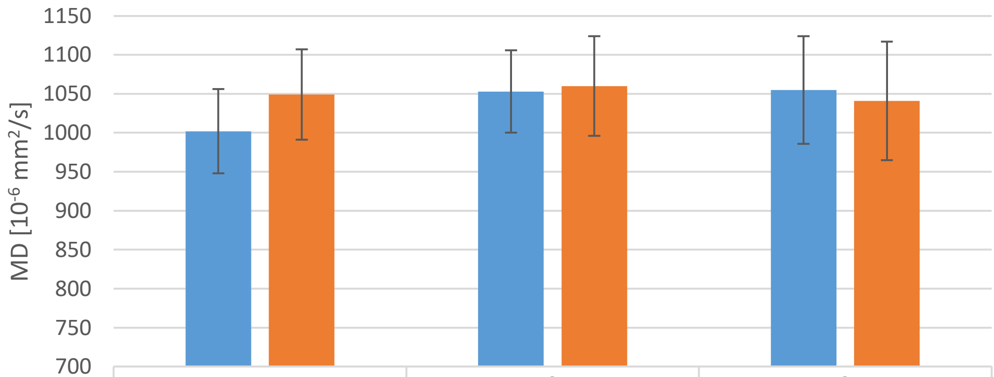

	1	2	3
CON	1002	1053	1055
HTN	1049	1060	1041

Axial diffusivity

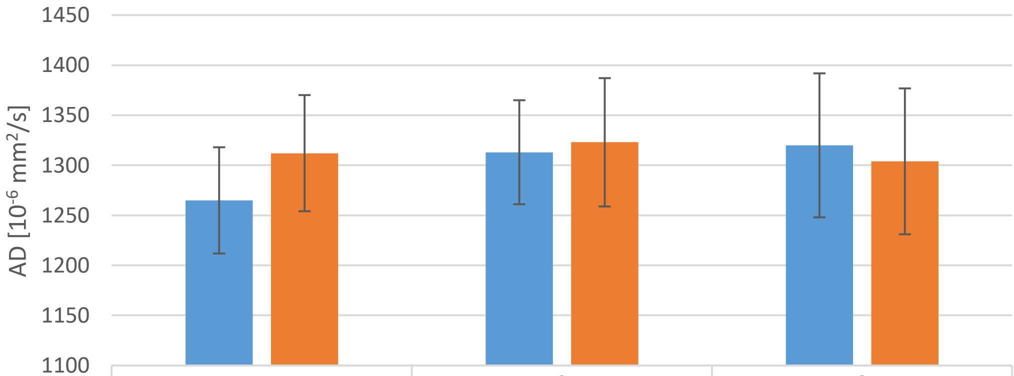

	1	2	3
CON	1265	1313	1320
HTN	1312	1323	1304

Left hemisphere white matter

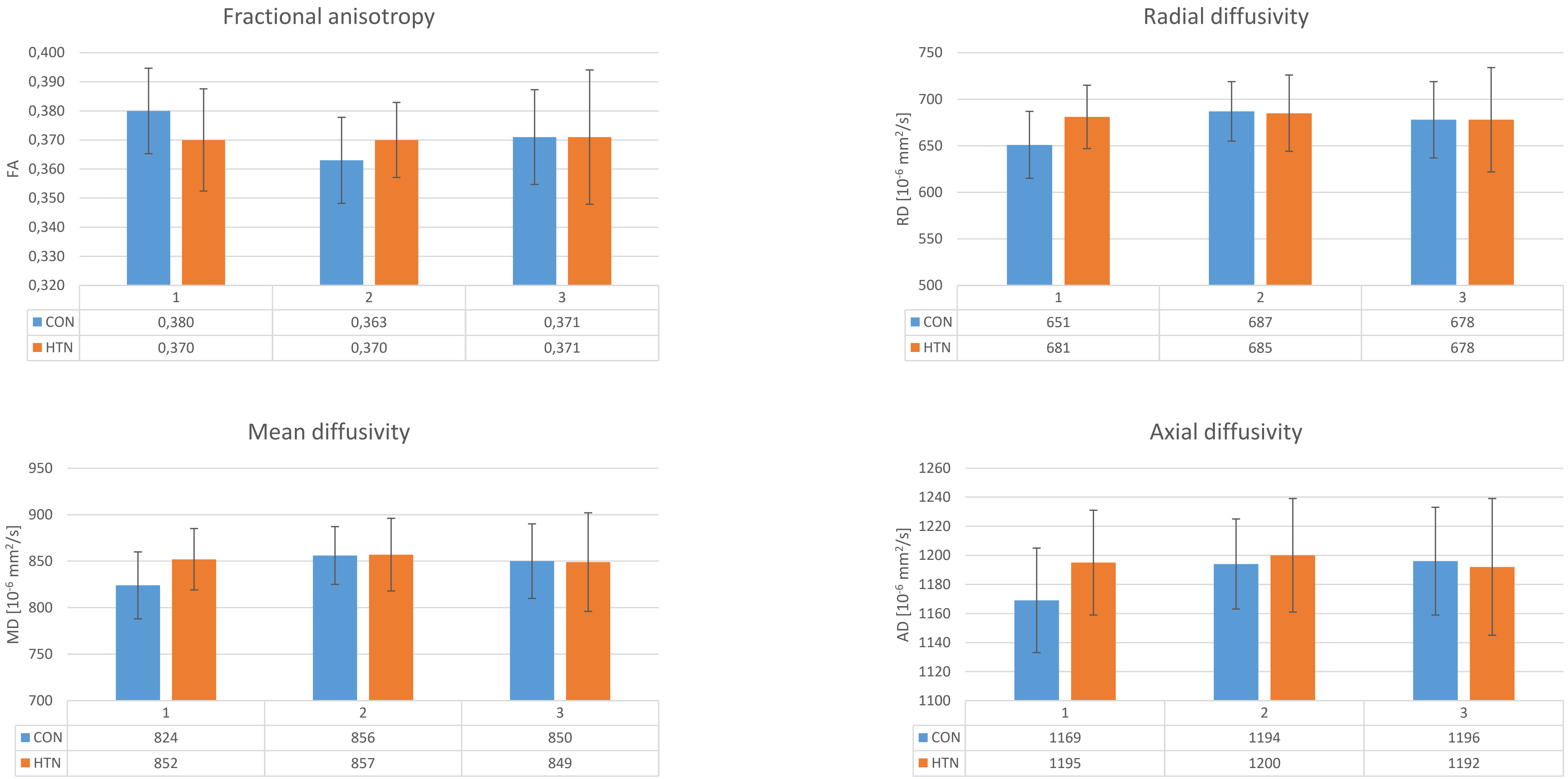

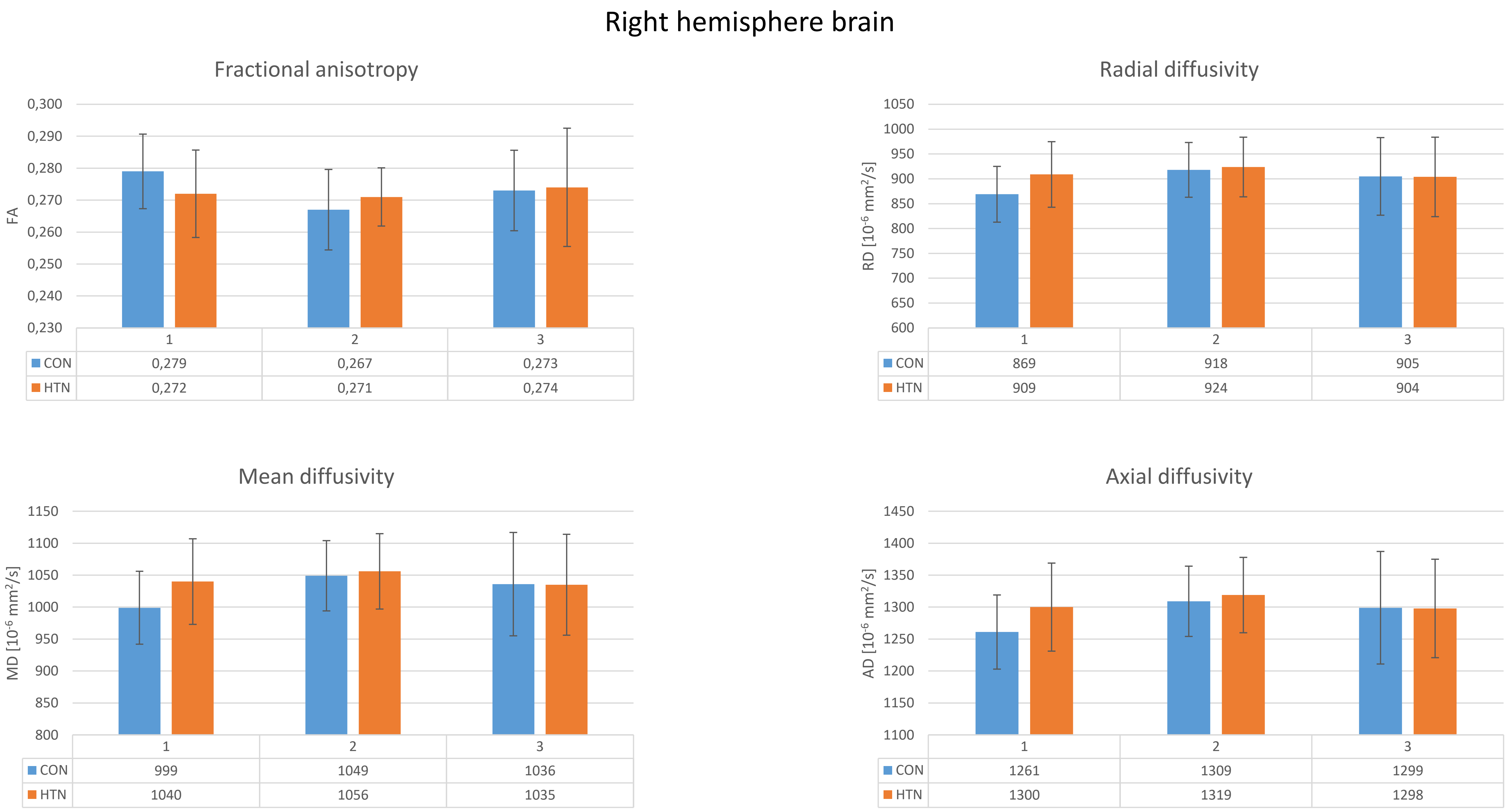
Right hemisphere brain
Fractional anisotropy
FA
0,300
0,290
0,280
0,270
0,260
0,250
0,240
0,230
1
2
3
CON 0,279 0,267 0,273
HTN 0,272 0,271 0,274
Radial diffusivity
RD [10-6 mm2/s]
1050
1000
950
900
850
800
750
700
650
600
1
2
3
CON 869 918 905
HTN 909 924 904
Mean diffusivity
MD [10-6 mm2/s]
1150
1100
1050
1000
950
900
850
800
1
2
3
CON 999 1049 1036
HTN 1040 1056 1035
Axial diffusivity
AD [10-6 mm2/s]
1450
1400
1350
1300
1250
1200
1150
1100
1
2
3
CON 1261 1309 1299
HTN 1300 1319 1298

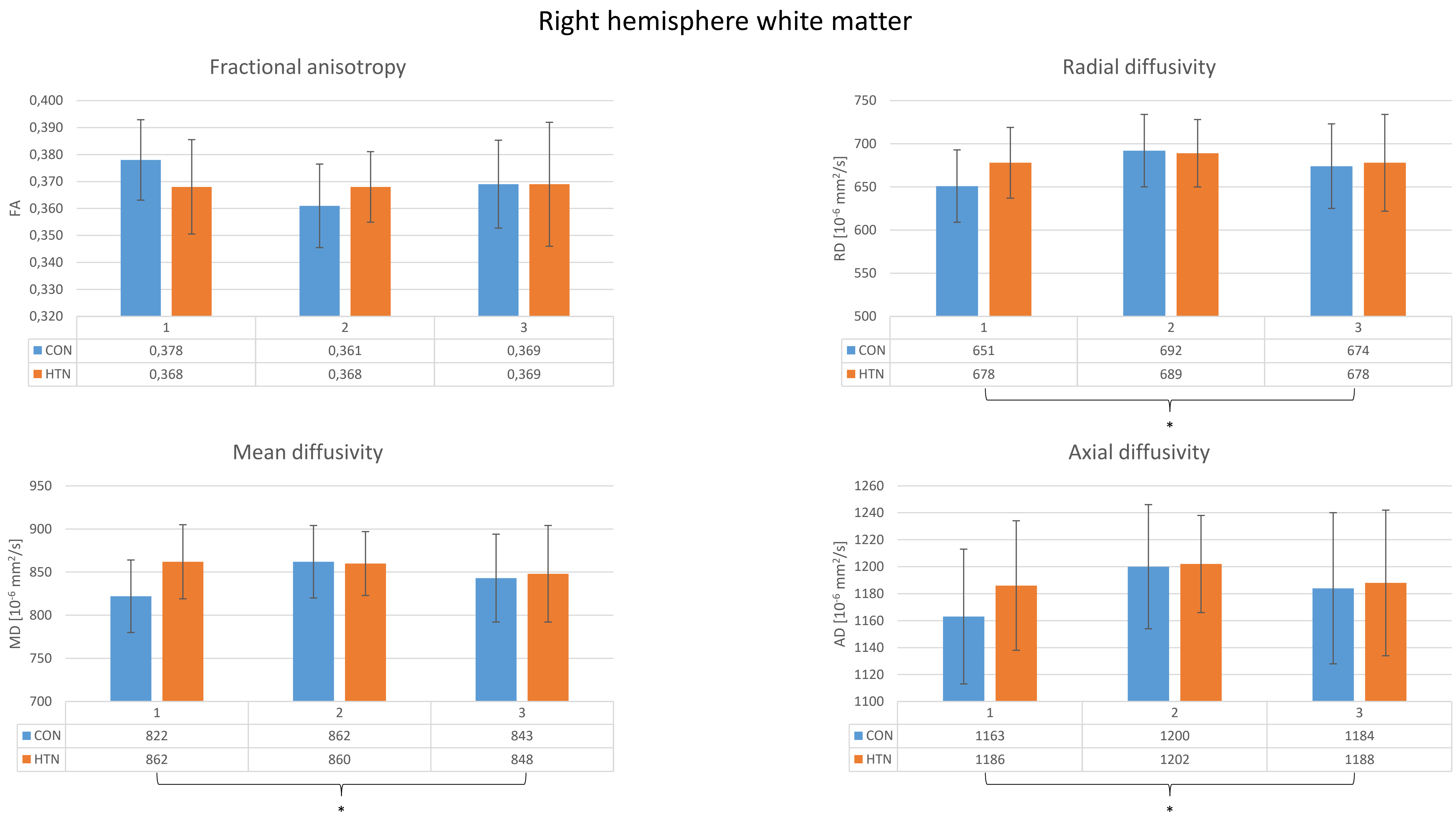
Right hemisphere white matter
Fractional anisotropy
FA
0,400
0,390
0,380
0,370
0,360
0,350
0,340
0,330
0,320
1
2
3
CON 0,378 0,361 0,369
HTN 0,368 0,368 0,369
Radial diffusivity
RD [10^-6 mm^2/s]
750
700
650
600
550
500
1
2
3
CON 651 692 674
HTN 678 689 678
*
Mean diffusivity
MD [10^-6 mm^2/s]
950
900
850
800
750
700
1
2
3
CON 822 862 843
HTN 862 860 848
*
Axial diffusivity
AD [10^-6 mm^2/s]
1260
1240
1220
1200
1180
1160
1140
1120
1100
1
2
3
CON 1163 1200 1184
HTN 1186 1202 1188
*

L1 – Anterior thalamic radiation left

Fractional anisotropy

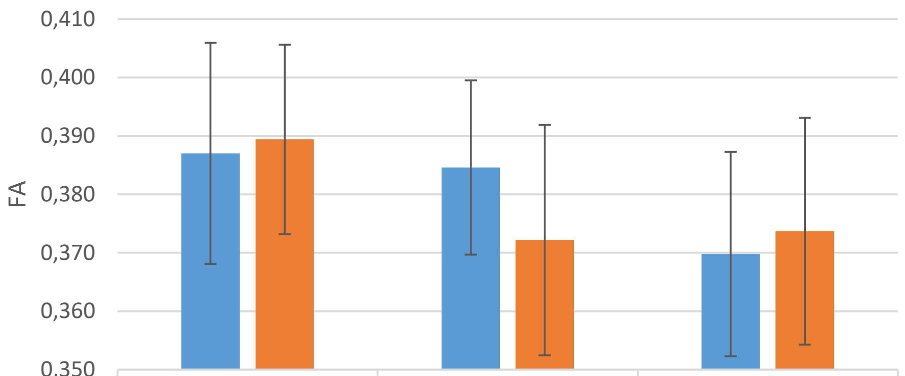

	1	2	3
CON	0,387	0,385	0,370
HTN	0,389	0,372	0,374

***

Radial diffusivity

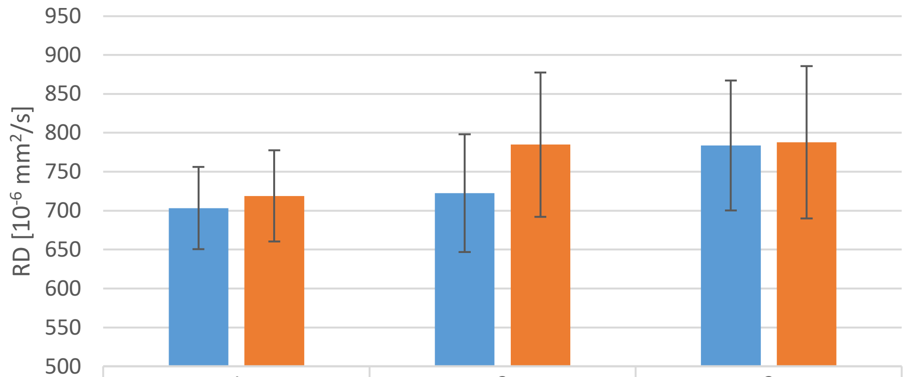

	1	2	3
CON	703	723	784
HTN	719	785	788

* (CON vs. HTN)

***

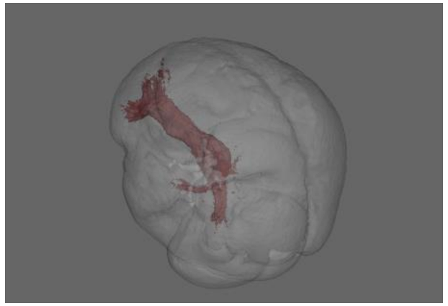

Control group

Patients with hypertension

Mean diffusivity

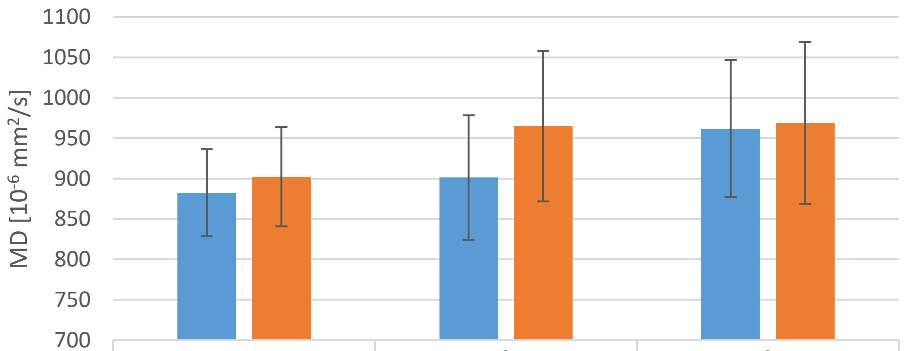

	1	2	3
CON	882	901	962
HTN	902	965	969

* (CON vs. HTN)

***

Axial diffusivity

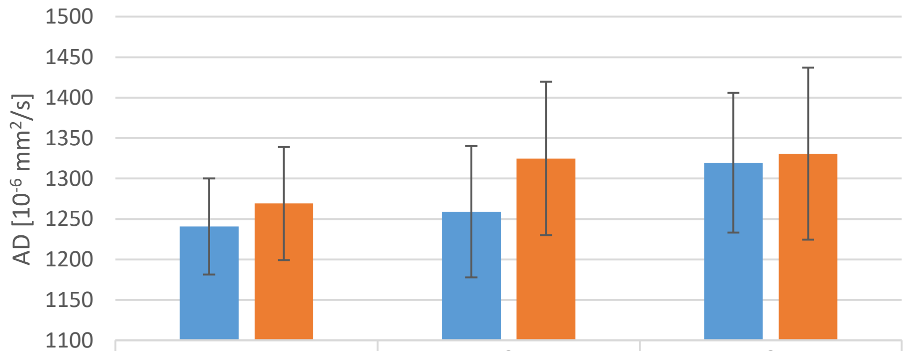

	1	2	3
CON	1241	1259	1320
HTN	1269	1325	1331

* (CON vs. HTN)

**

R1 – Anterior thalamic radiation right

Fractional anisotropy

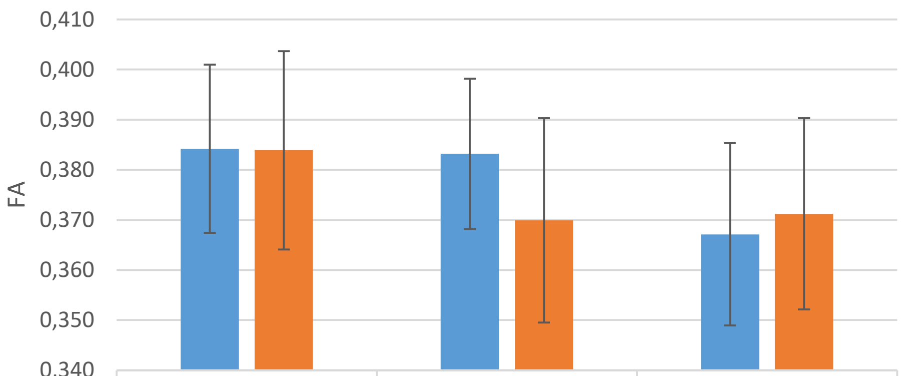

	1	2	3
CON	0,384	0,383	0,367
HTN	0,3839	0,3699	0,3712

***

Radial diffusivity

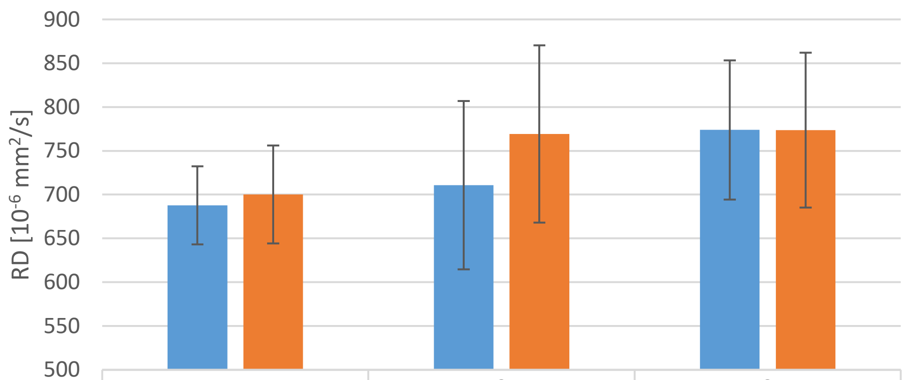

	1	2	3
CON	688	711	774
HTN	700	769	774

***

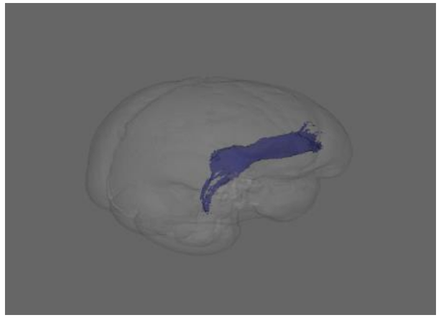

Control group

Patients with hypertension

Mean diffusivity

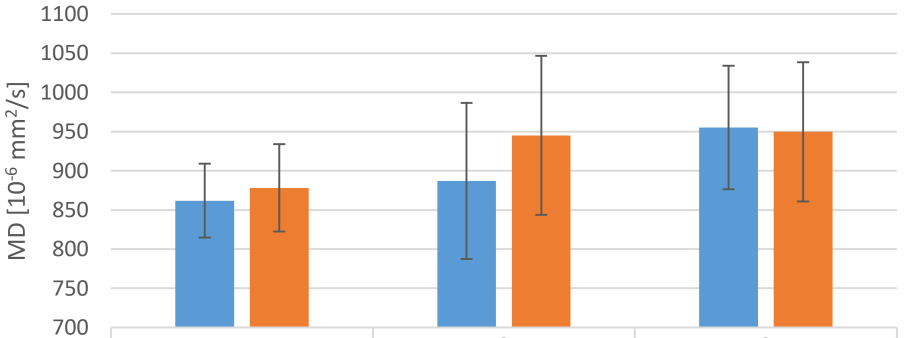

	1	2	3
CON	862	887	955
HTN	878	945	950

***

Axial diffusivity

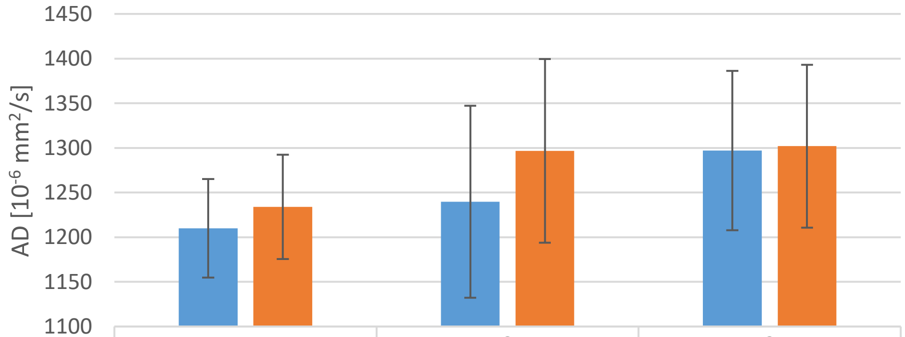

	1	2	3
CON	1210	1240	1297
HTN	1234	1297	1302

***

L2 – Corticospinal tract left

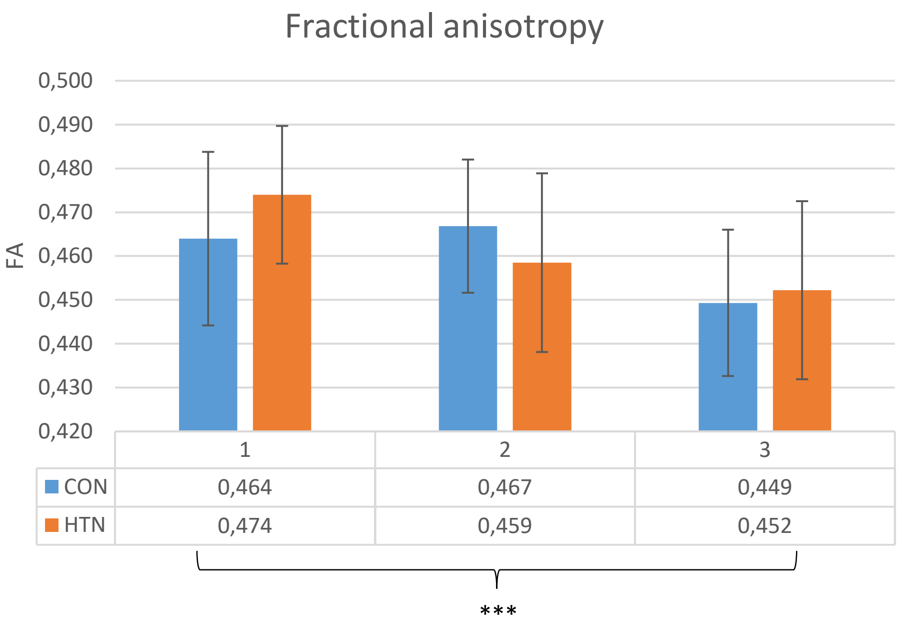

	1	2	3
CON	0,464	0,467	0,449
HTN	0,474	0,459	0,452

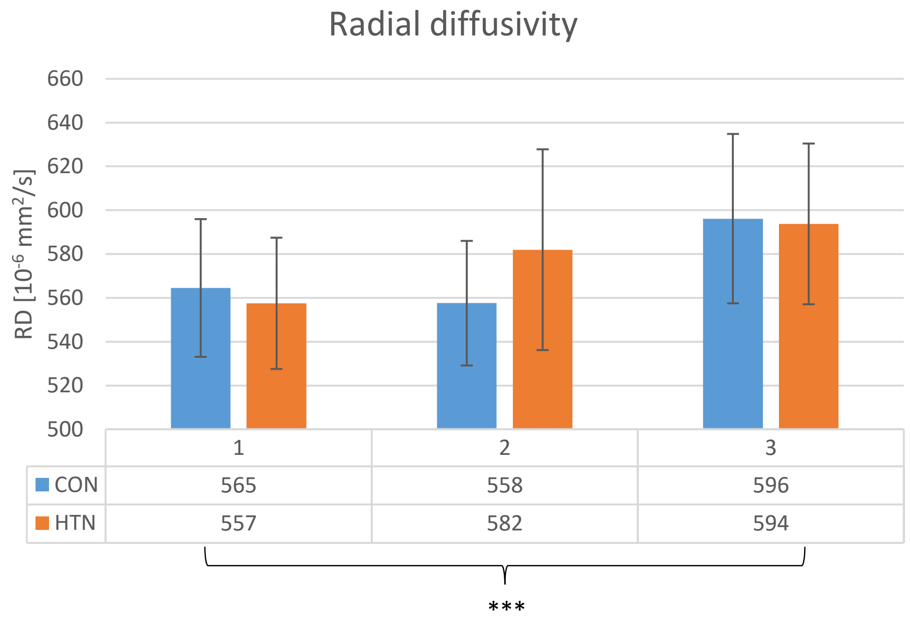

	1	2	3
CON	565	558	596
HTN	557	582	594

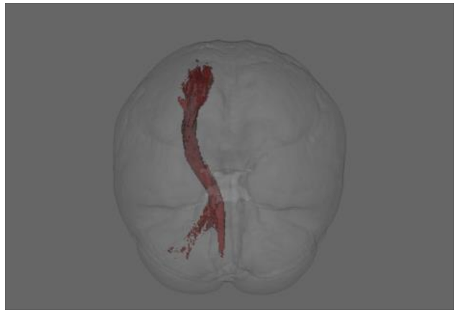

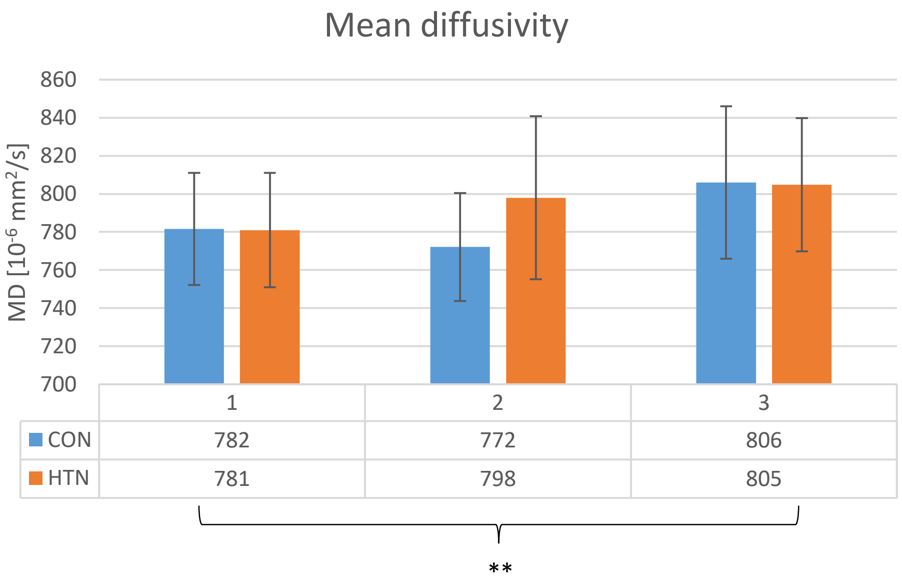

	1	2	3
CON	782	772	806
HTN	781	798	805

Control group

Patients with hypertension

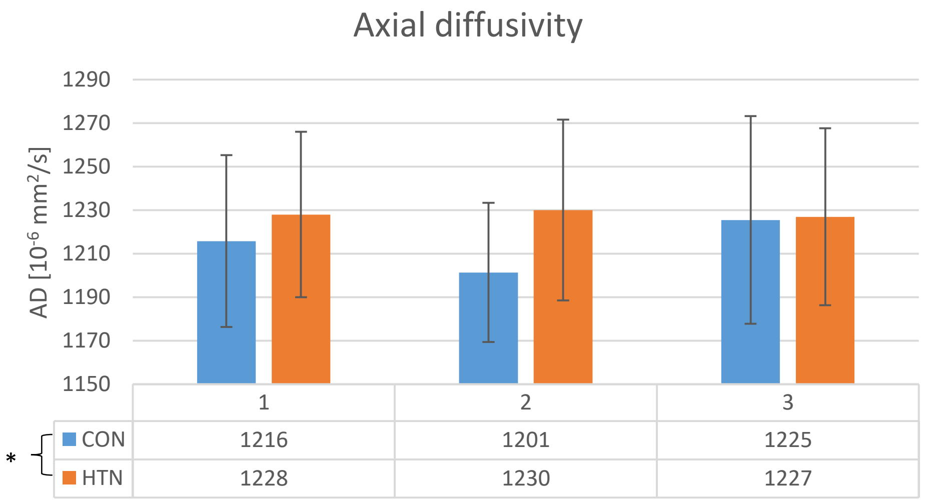

	1	2	3
CON	1216	1201	1225
HTN	1228	1230	1227

R2 – Corticospinal tract right

Fractional anisotropy

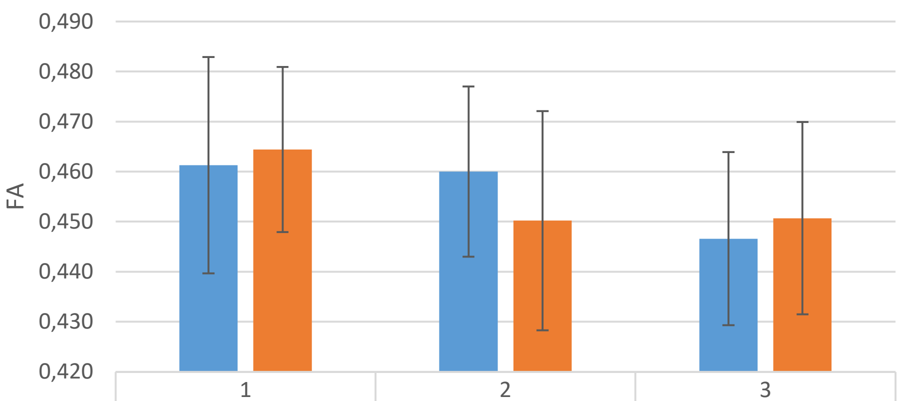

	1	2	3
CON	0,461	0,460	0,447
HTN	0,464	0,450	0,451

**

Radial diffusivity

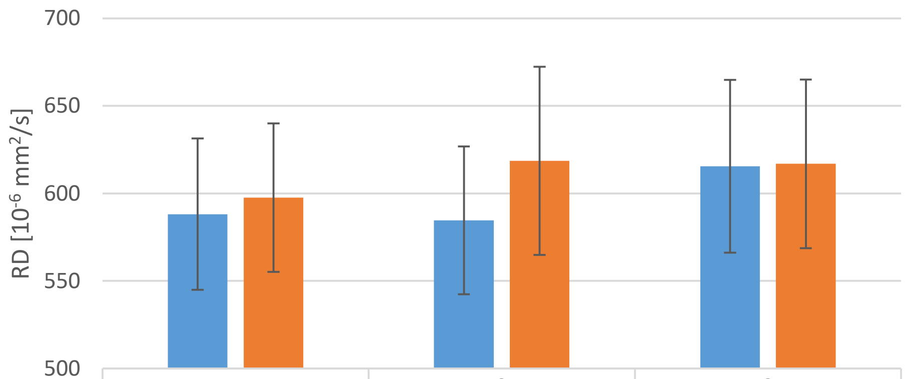

	1	2	3
CON	588	585	615
HTN	598	619	617

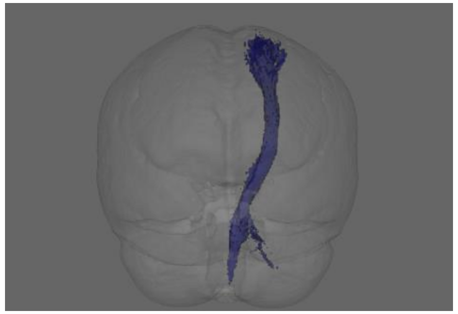

Control group

Patients with hypertension

Mean diffusivity

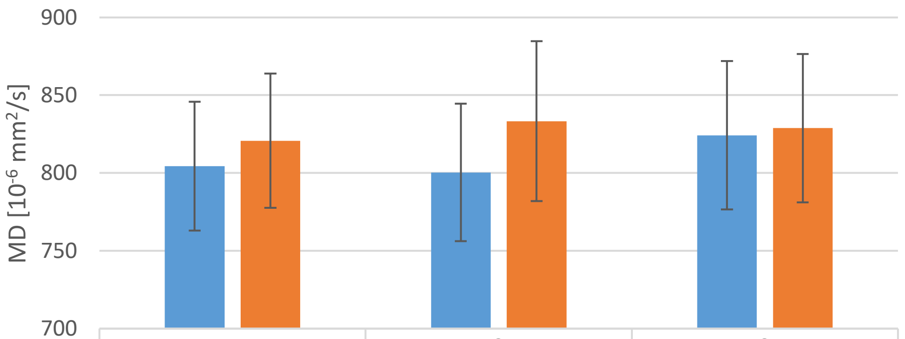

	1	2	3
CON	804	800	824
HTN	821	833	829

*

Axial diffusivity

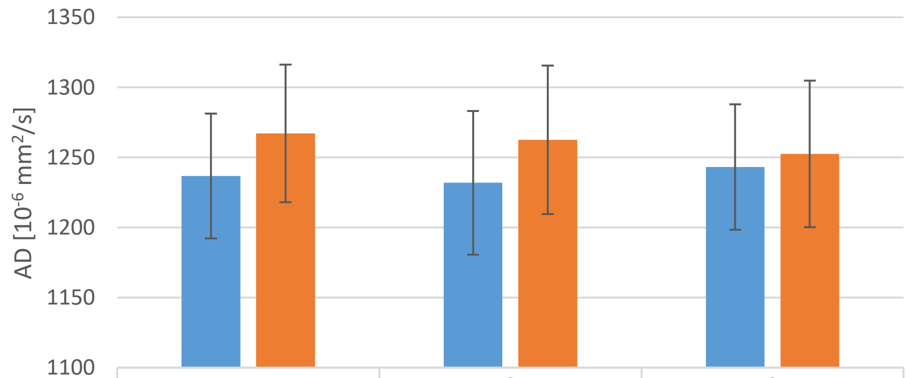

	1	2	3
CON	1237	1232	1243
HTN	1267	1263	1253

**

FX - Fornix

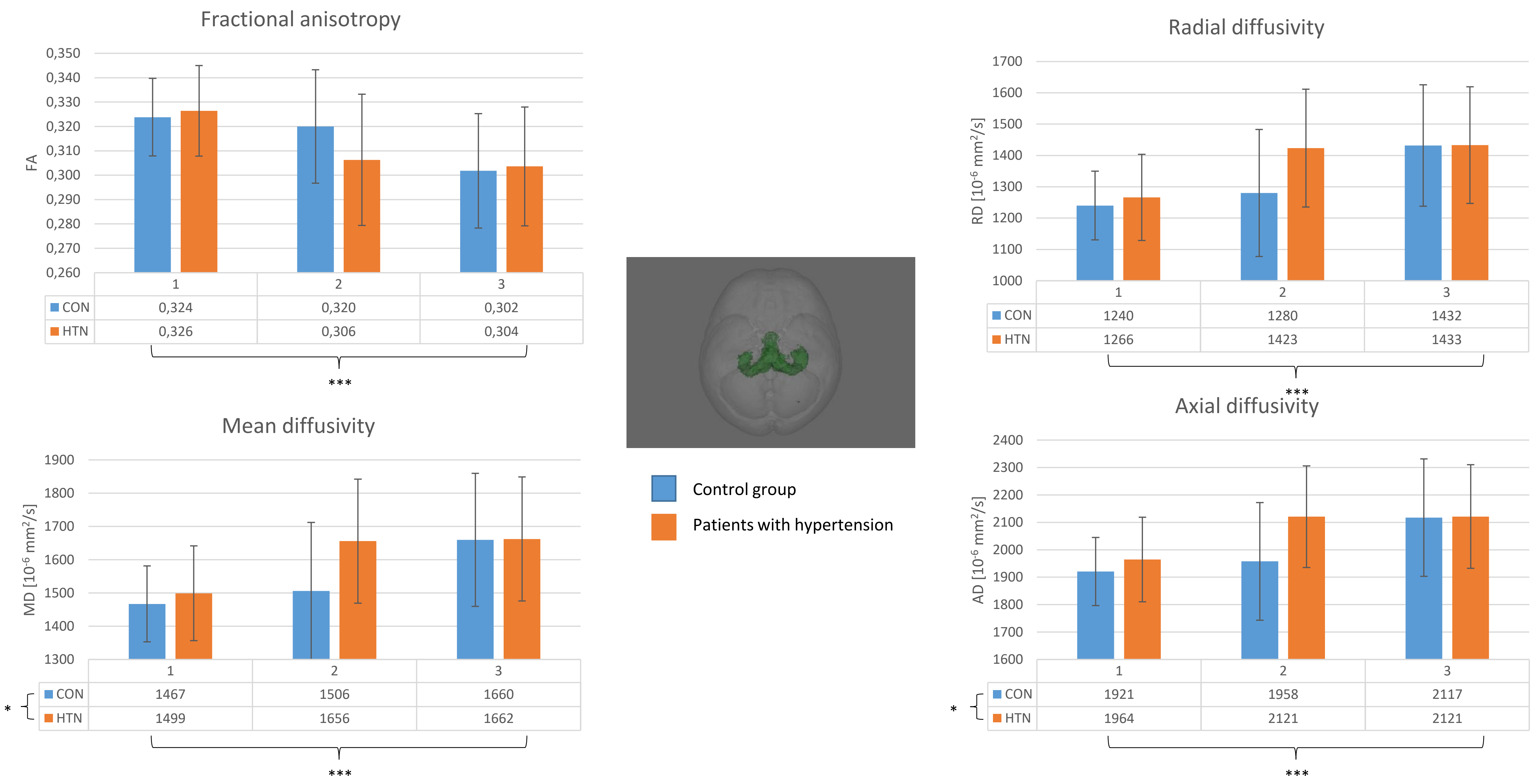

Fractional anisotropy

	1	2	3
CON	0,324	0,320	0,302
HTN	0,326	0,306	0,304

Mean diffusivity

	1	2	3
CON	1467	1506	1660
HTN	1499	1656	1662

Radial diffusivity

	1	2	3
CON	1240	1280	1432
HTN	1266	1423	1433

Axial diffusivity

	1	2	3
CON	1921	1958	2117
HTN	1964	2121	2121

CC – Corpus callosum

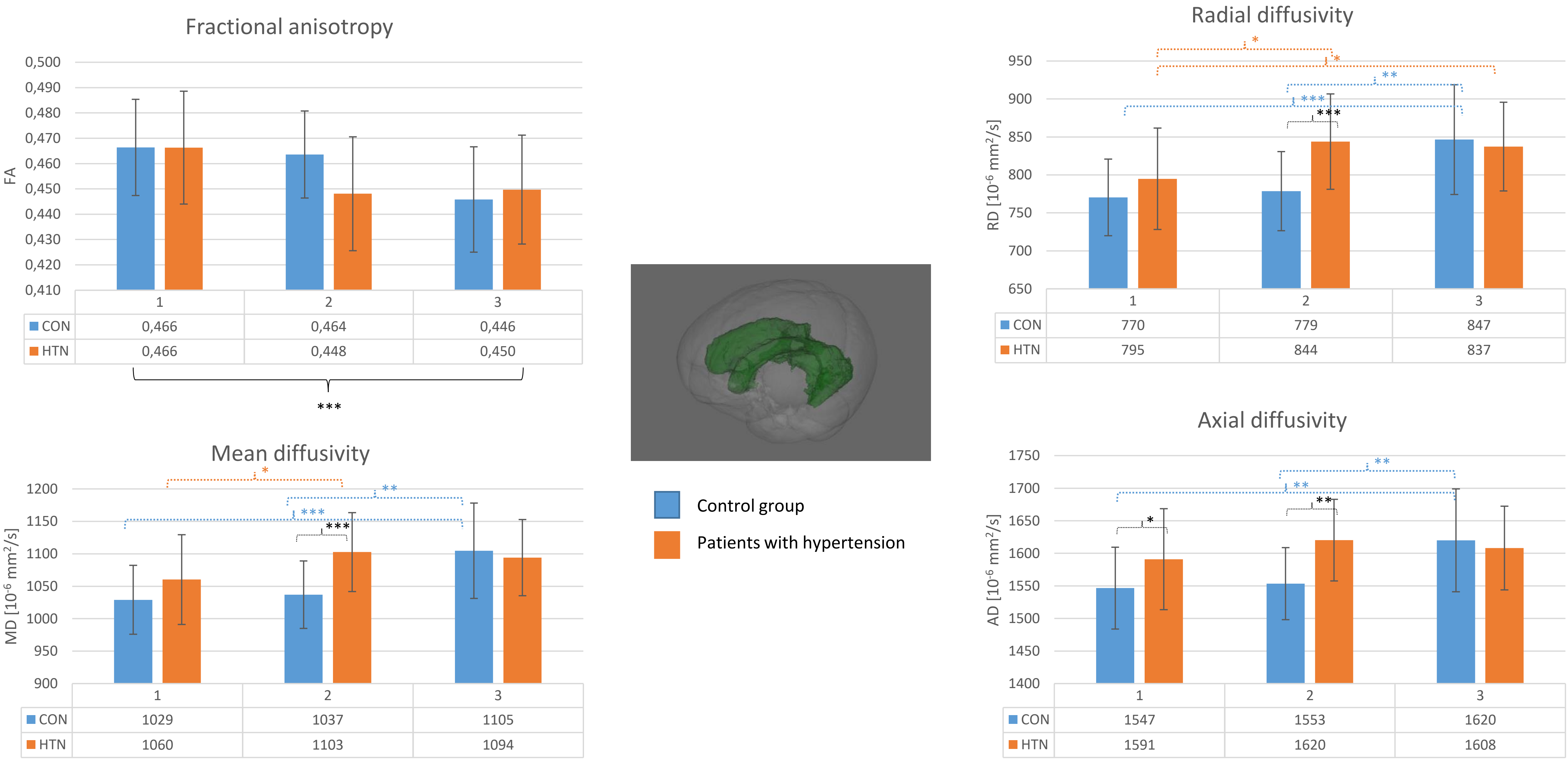

L3 – Cingulum (hippocampus) left

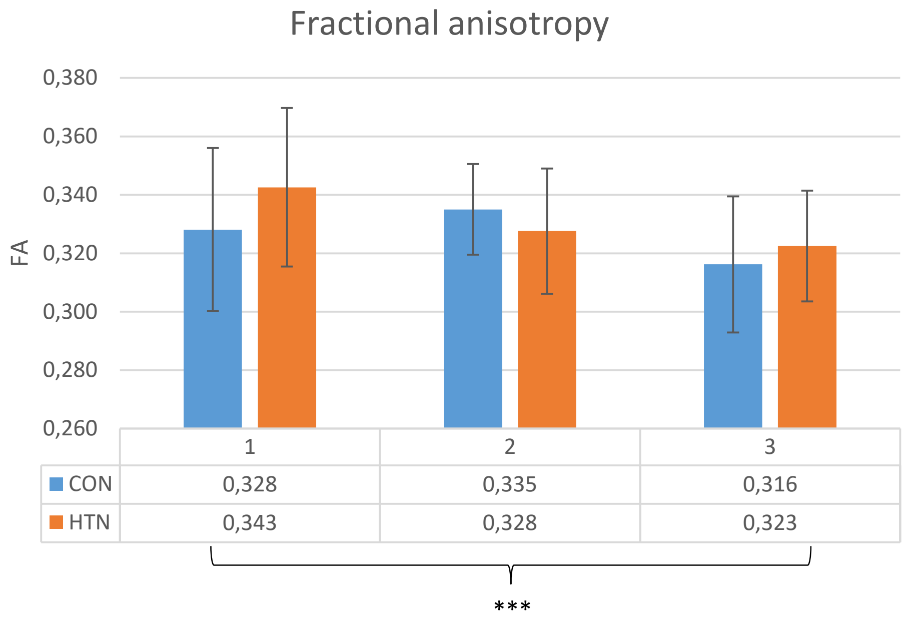

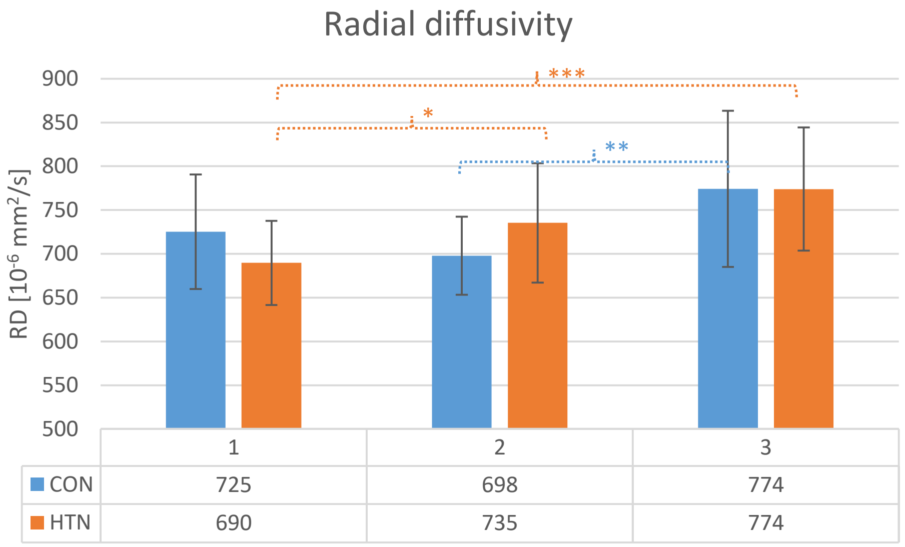

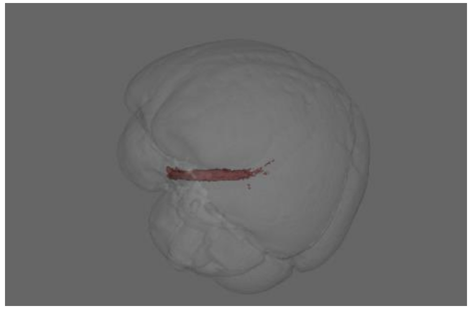

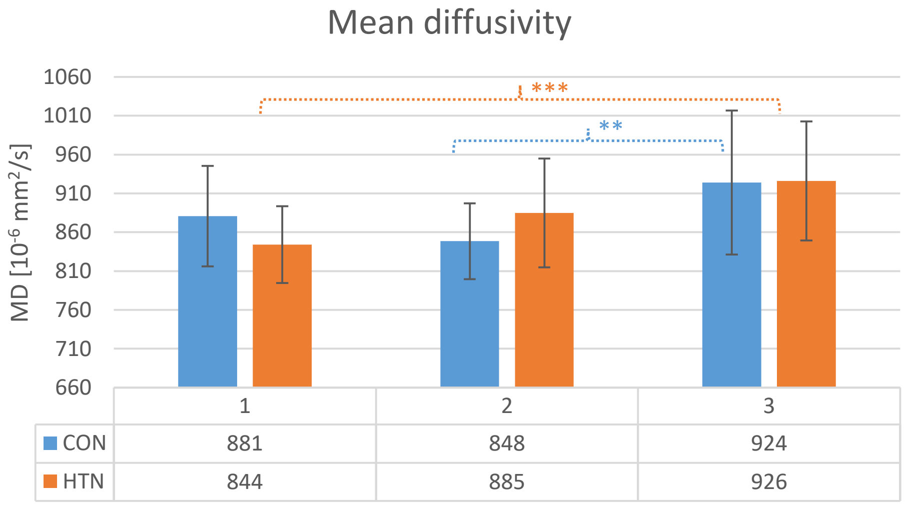

Control group

Patients with hypertension

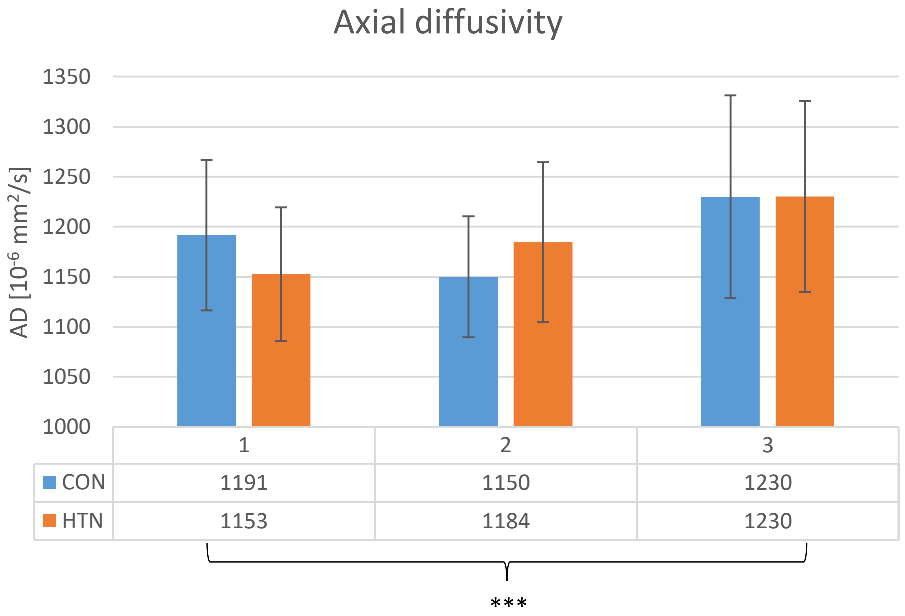

R3 – Cingulum (hippocampus) right

Fractional anisotropy

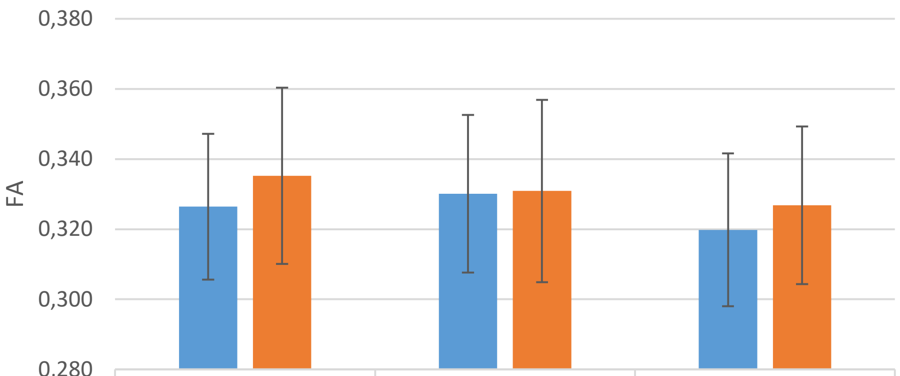

	1	2	3
CON	0,326	0,330	0,320
HTN	0,335	0,331	0,327

Radial diffusivity

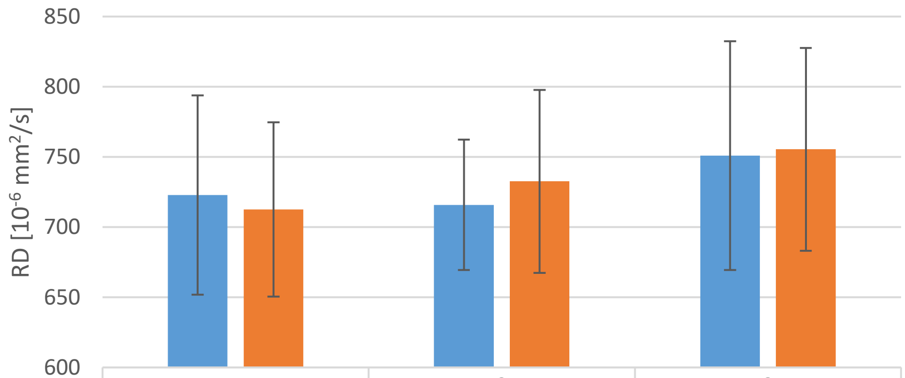

	1	2	3
CON	723	716	751
HTN	713	733	755

*

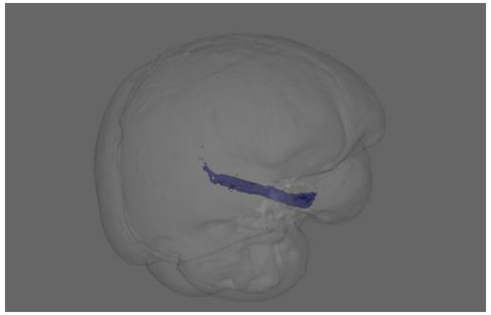

Control group

Patients with hypertension

Mean diffusivity

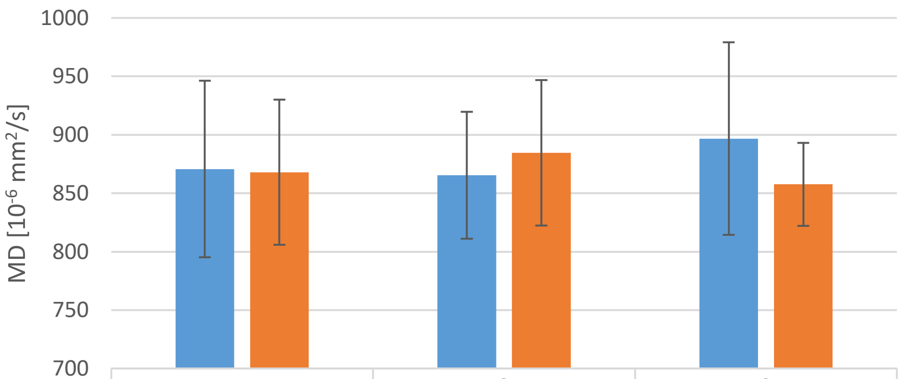

	1	2	3
CON	871	865	897
HTN	868	885	858

Axial diffusivity

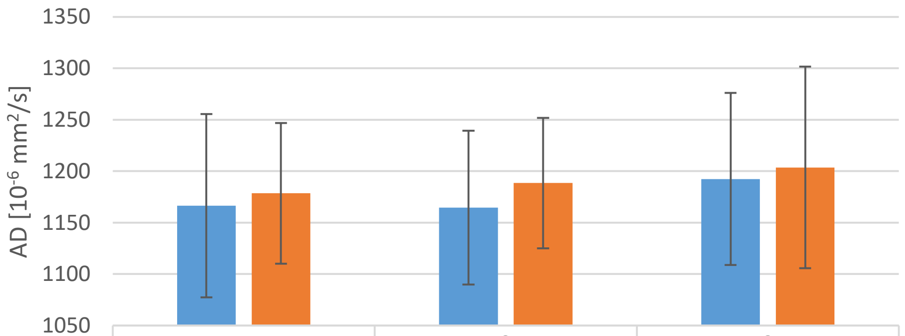

	1	2	3
CON	1166	1165	1192
HTN	1178,588653	1188,461168	1203,64162

L4 – Cingulum (cingulate gyrus) left

Fractional anisotropy

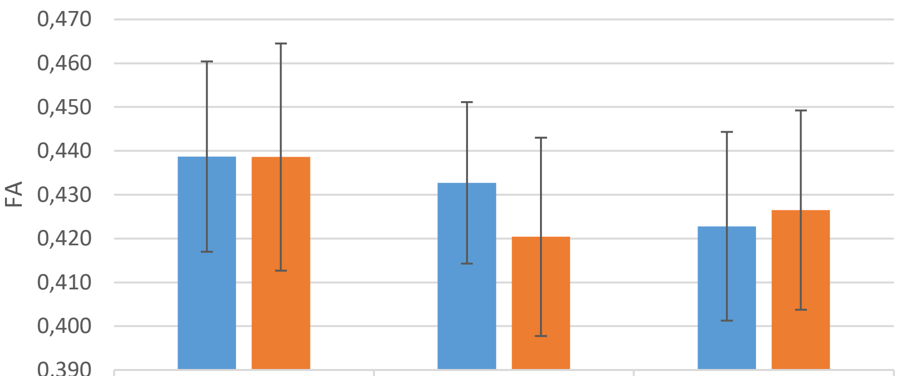

	1	2	3
CON	0,439	0,433	0,423
HTN	0,439	0,420	0,427

**

Radial diffusivity

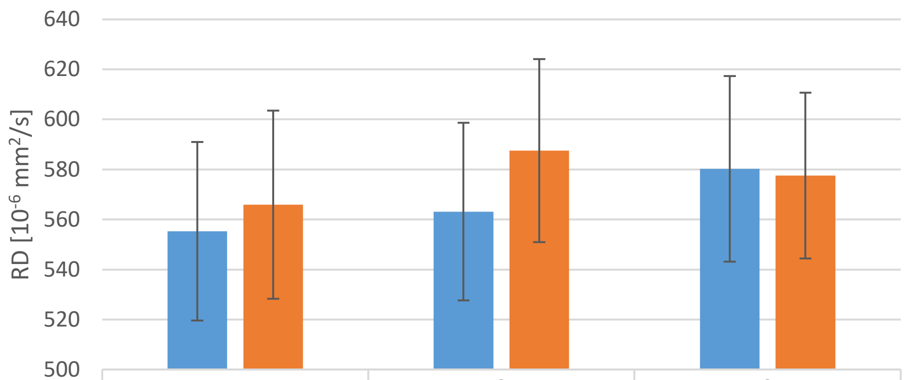

	1	2	3
CON	555	563	580
HTN	566	588	578

*

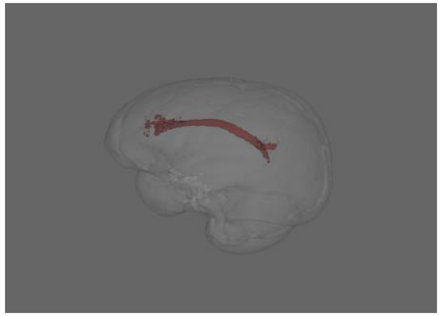

Control group

Patients with hypertension

Mean diffusivity

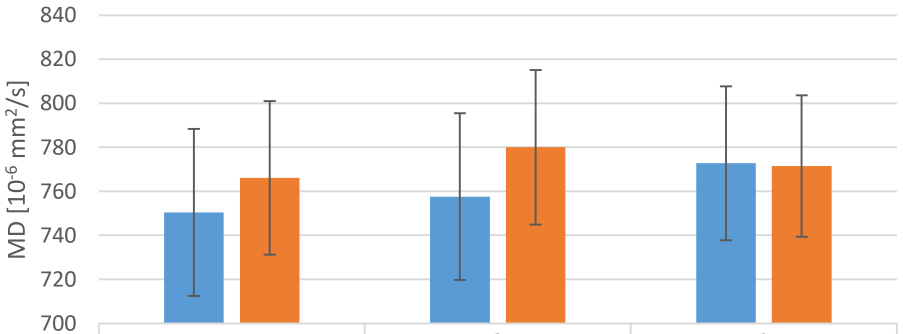

	1	2	3
CON	750	758	773
HTN	766	780	771

* (CON vs HTN)

Axial diffusivity

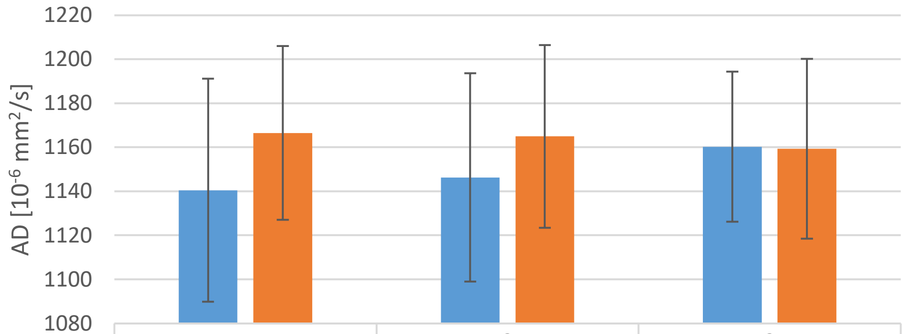

	1	2	3
CON	1140	1146	1160
HTN	1166	1165	1159

* (CON vs HTN)

R4 – Cingulum (cingulate gyrus) right

Fractional anisotropy

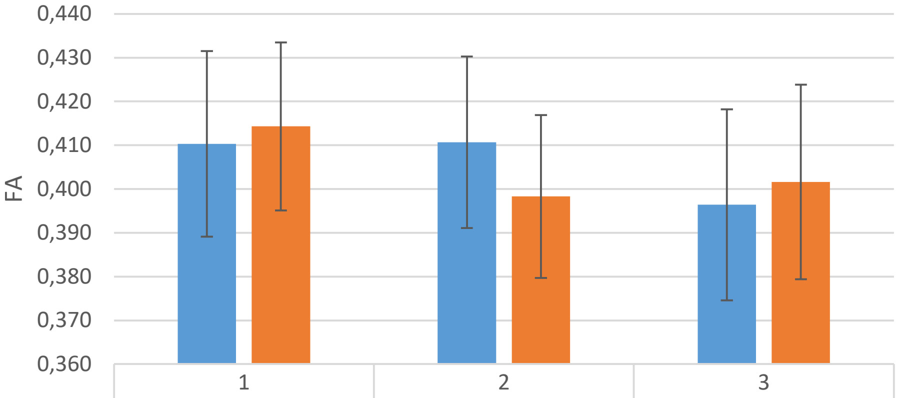

	1	2	3
CON	0,410	0,411	0,396
HTN	0,414	0,398	0,402

**

Radial diffusivity

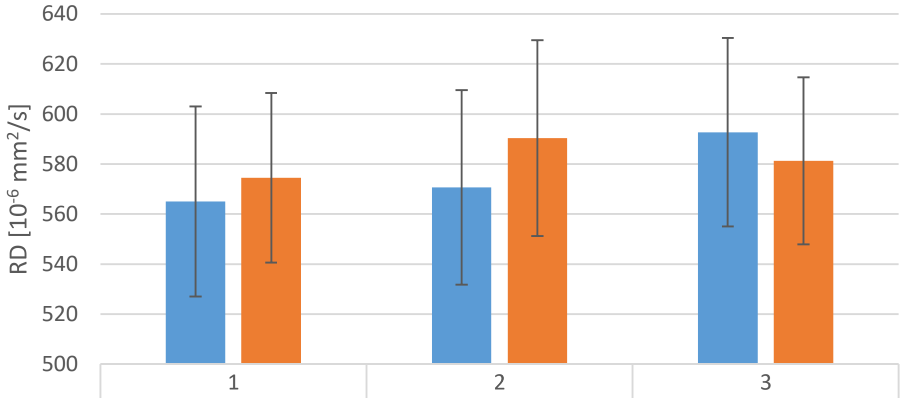

	1	2	3
CON	565	571	593
HTN	574	590	581

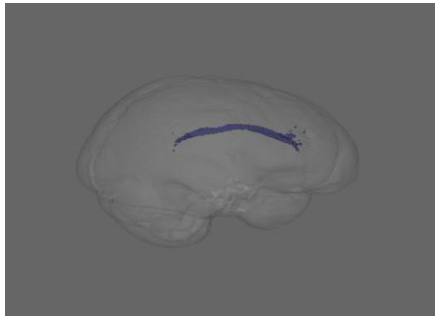

Control group

Patients with hypertension

Mean diffusivity

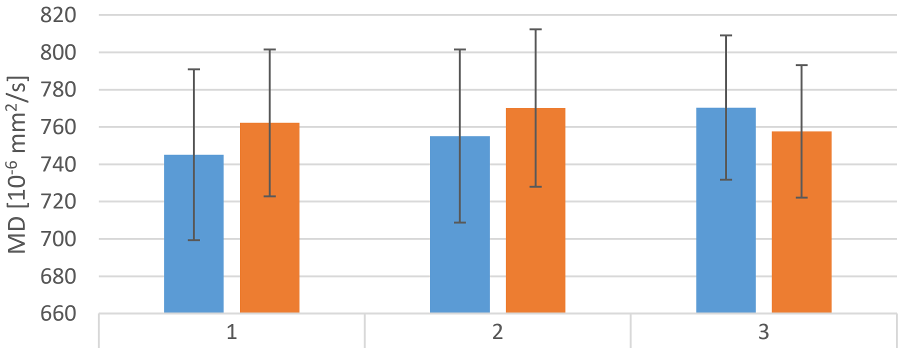

	1	2	3
CON	745	755	770
HTN	762	770	758

Axial diffusivity

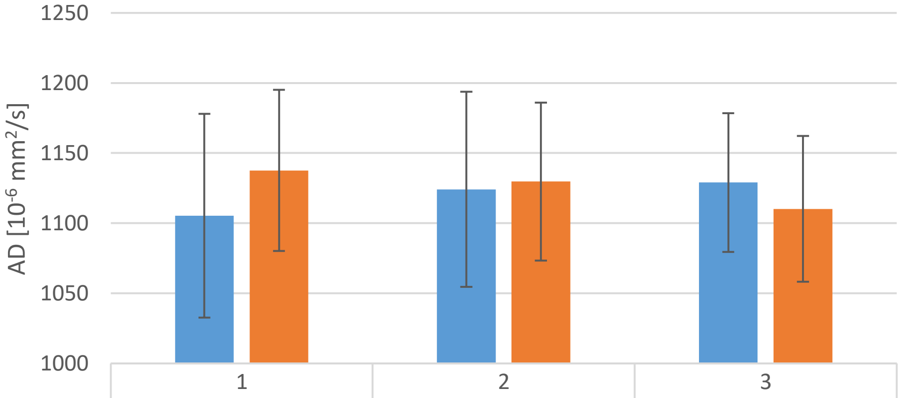

	1	2	3
CON	1105	1124	1129
HTN	1138	1130	1110

L5 – Inferior fronto-occipital fasciculus left

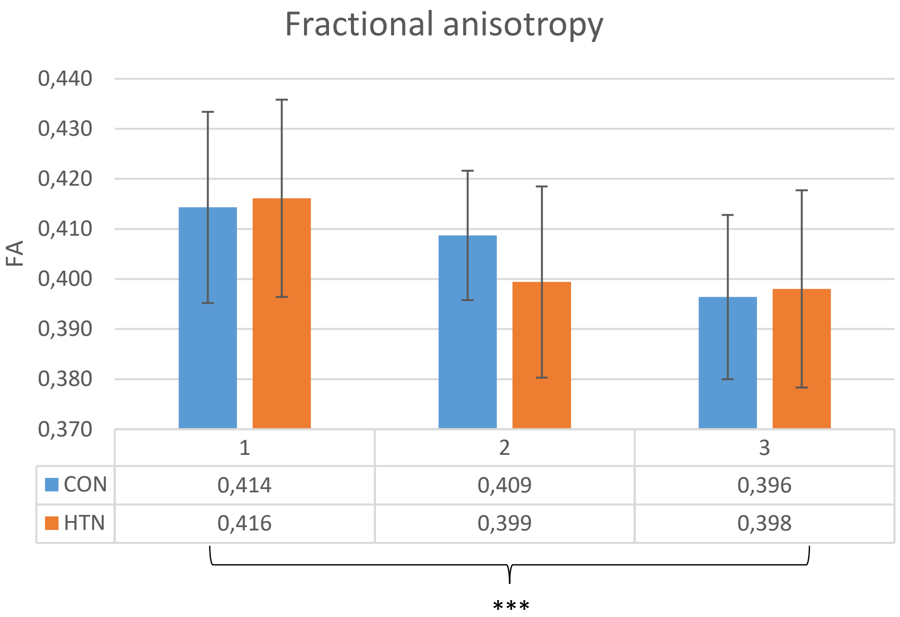

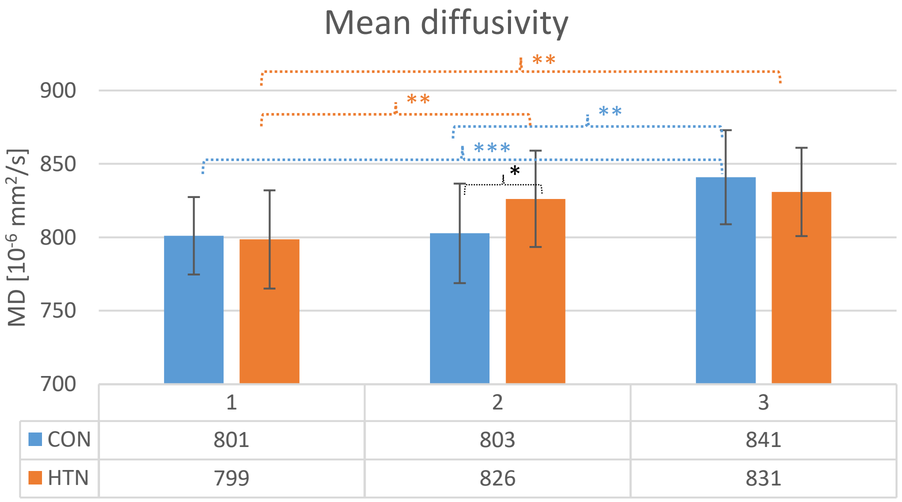

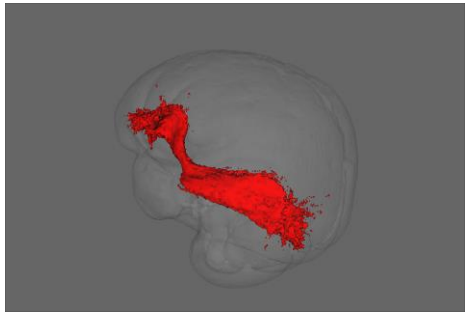

Control group

Patients with hypertension

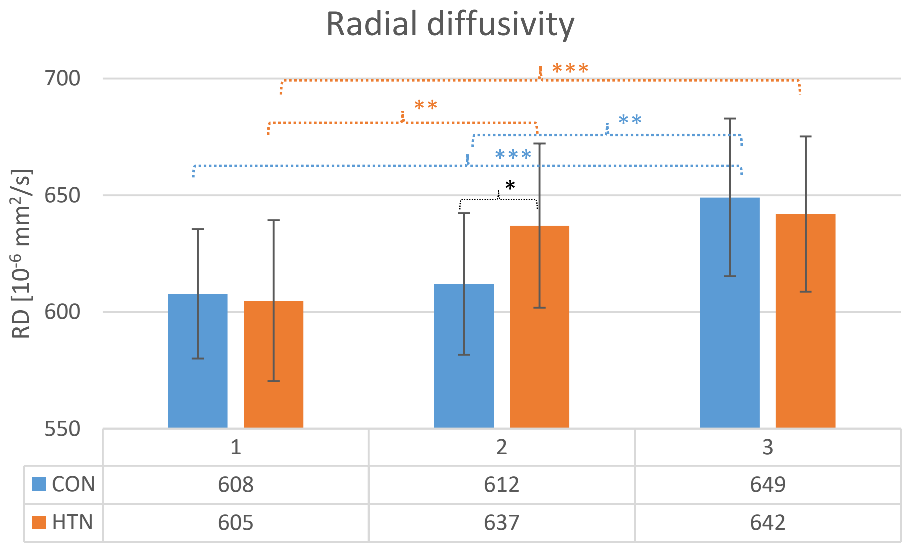

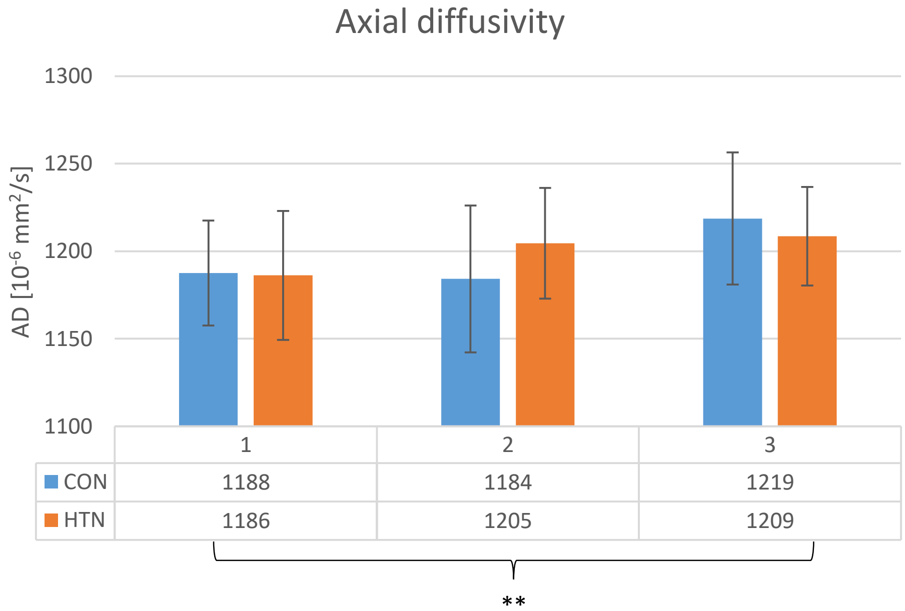

R5 – Inferior fronto-occipital fasciculus right

Fractional anisotropy

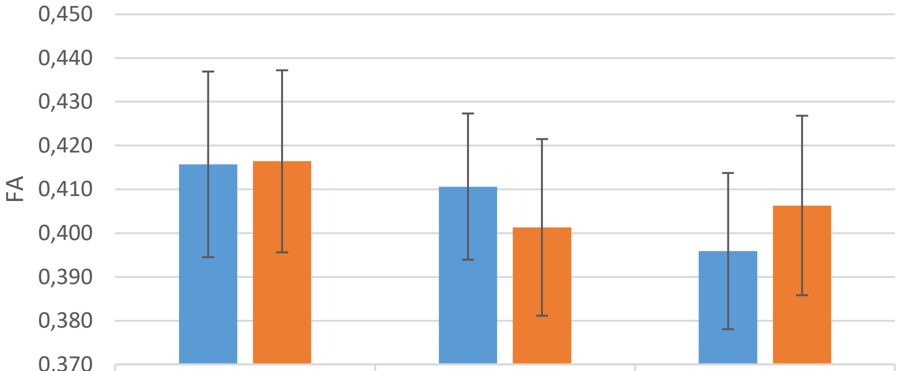

	1	2	3
CON	0,416	0,411	0,396
HTN	0,416	0,401	0,406

***

Radial diffusivity

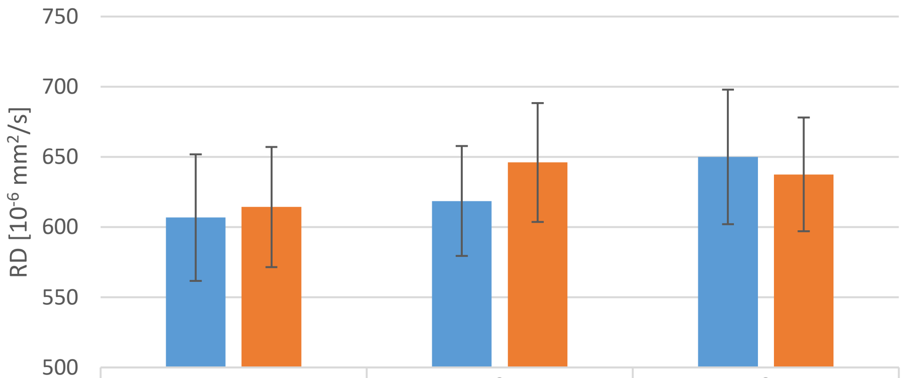

	1	2	3
CON	607	619	650
HTN	614	646	638

***

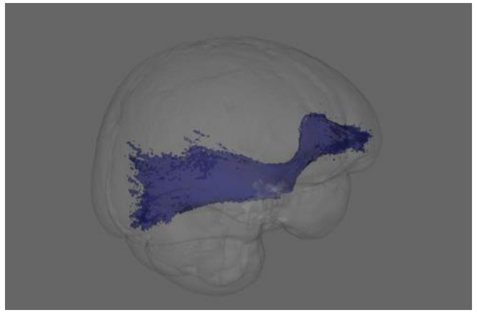

Control group

Patients with hypertension

Mean diffusivity

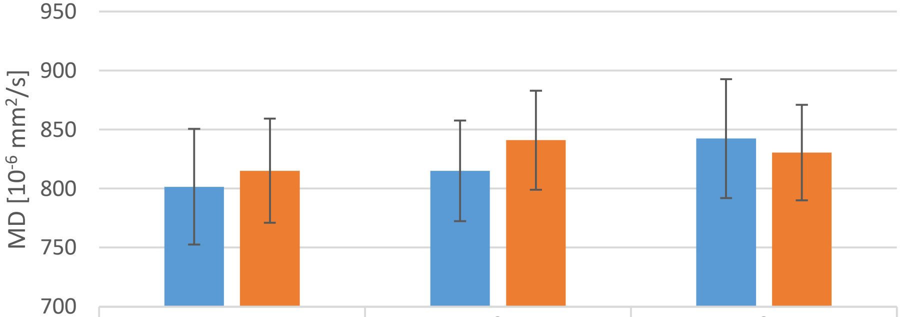

	1	2	3
CON	802	815	842
HTN	815	841	831

***

Axial diffusivity

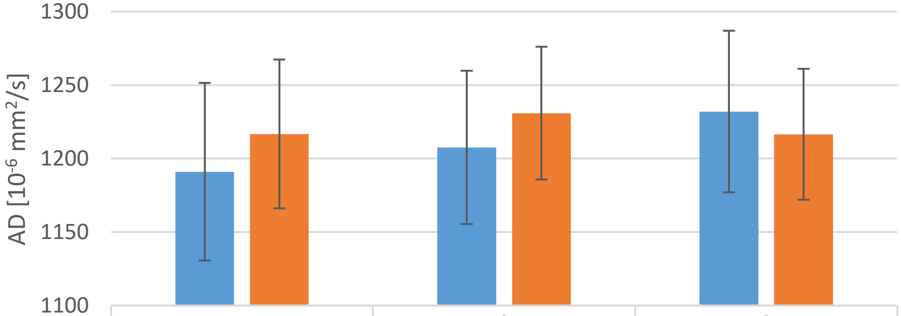

	1	2	3
CON	1191	1208	1232
HTN	1217	1231	1216

L6 – Inferior longitudinal fasciculus left

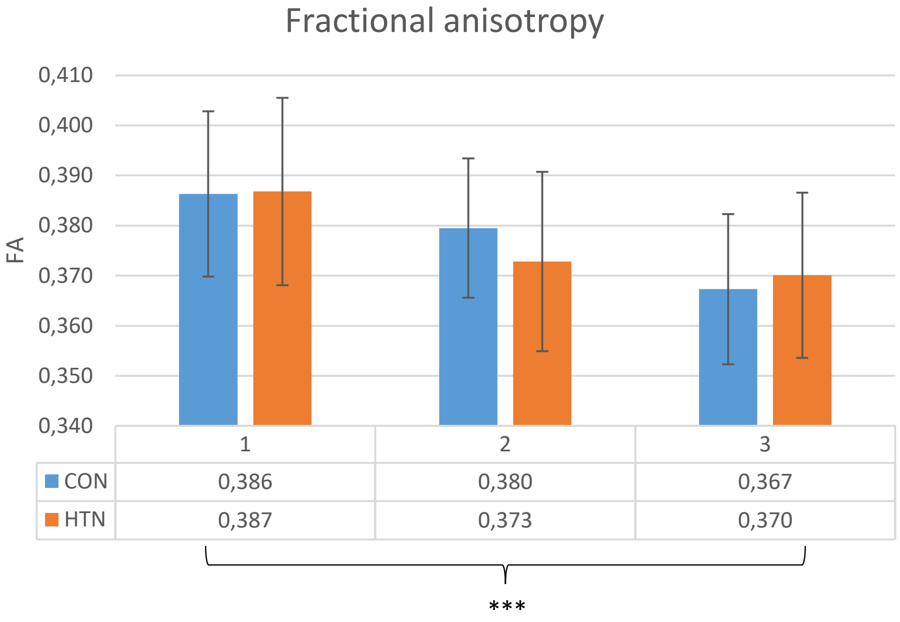

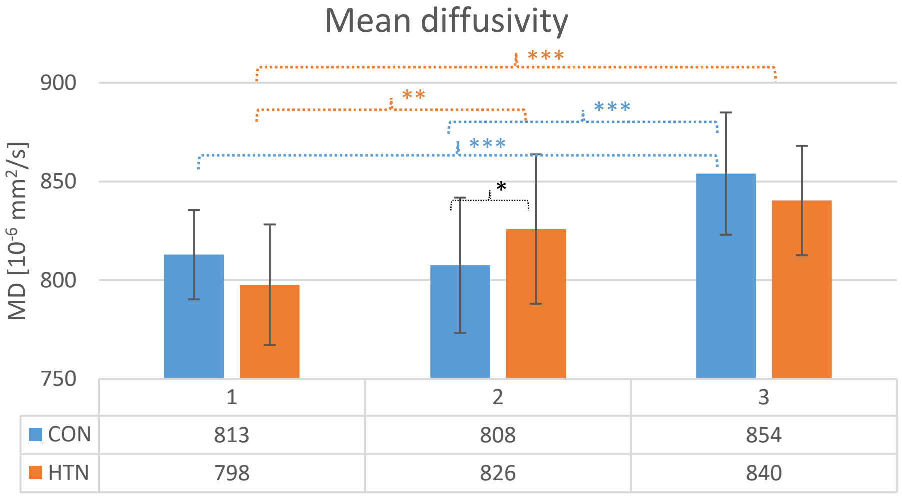

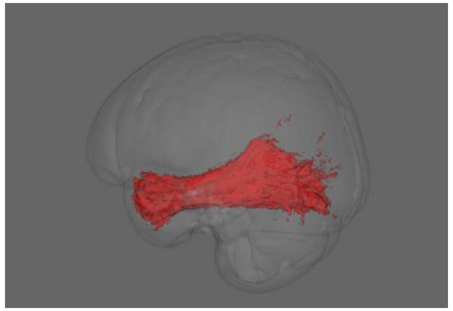

Control group

Patients with hypertension

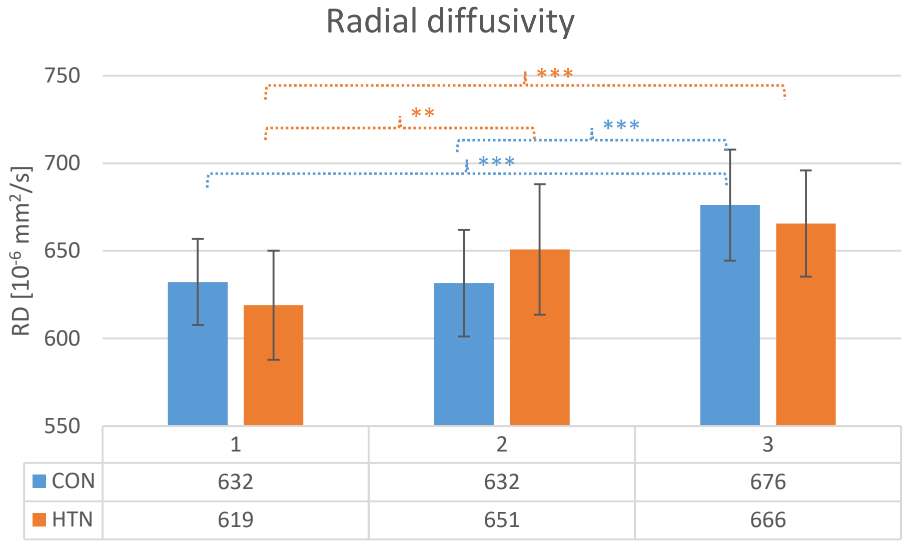

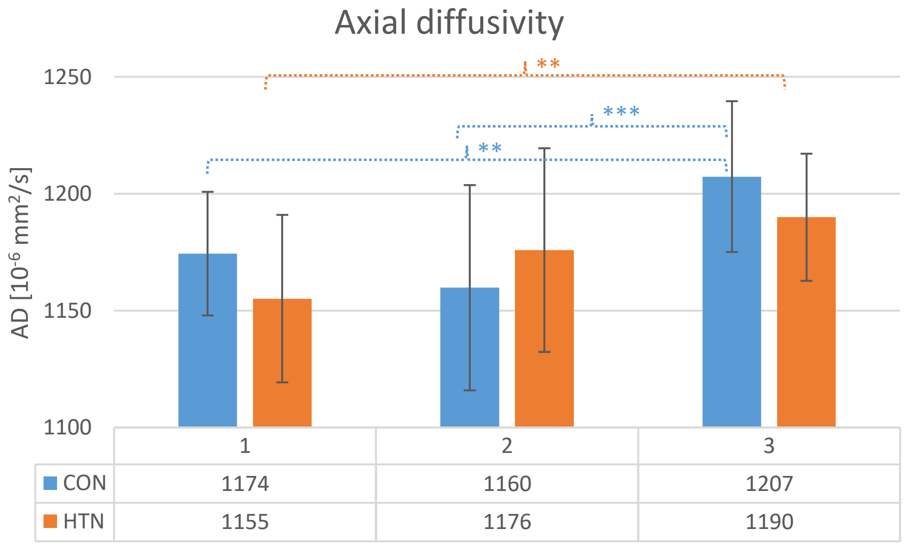

R6 – Inferior longitudinal fasciculus right

Fractional anisotropy

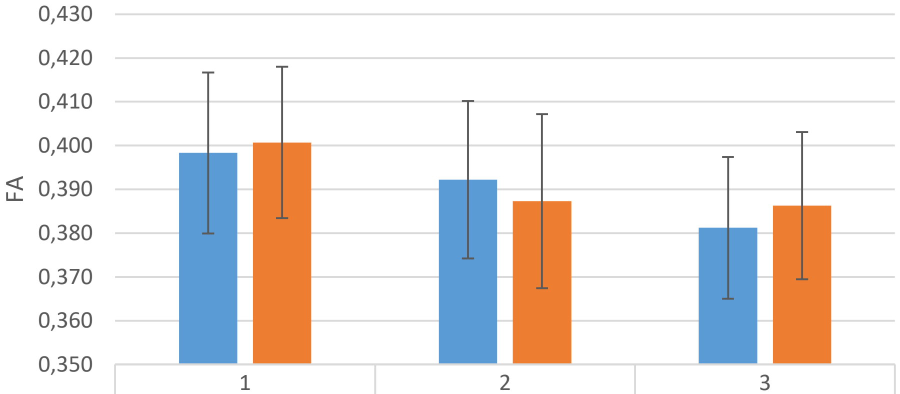

	1	2	3
CON	0,398	0,392	0,381
HTN	0,401	0,387	0,386

***

Radial diffusivity

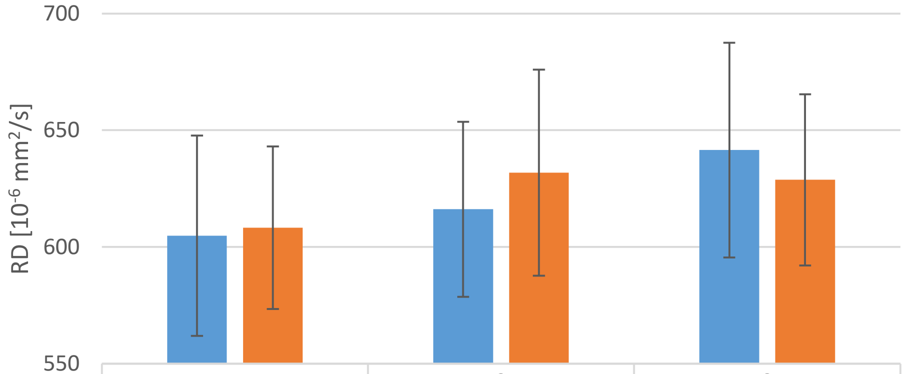

	1	2	3
CON	605	616	641
HTN	608	632	629

**

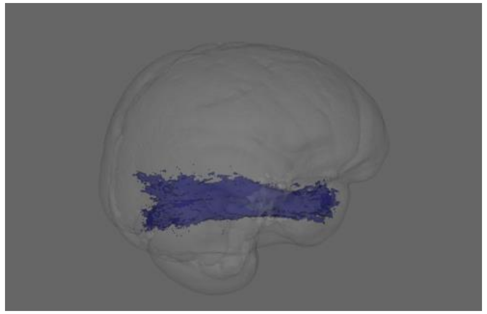

Control group

Patients with hypertension

Mean diffusivity

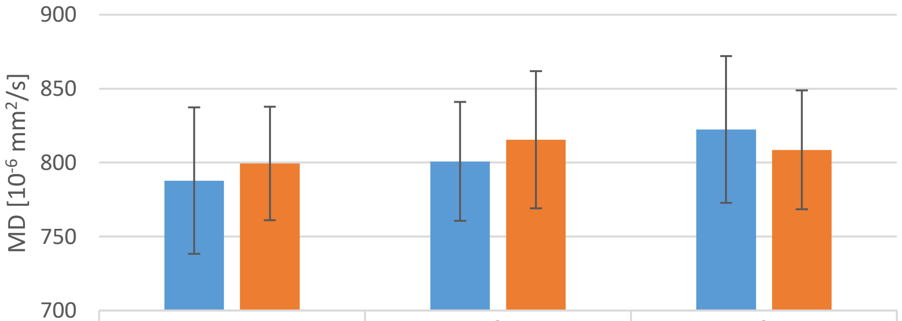

	1	2	3
CON	788	801	822
HTN	799	815	809

Axial diffusivity

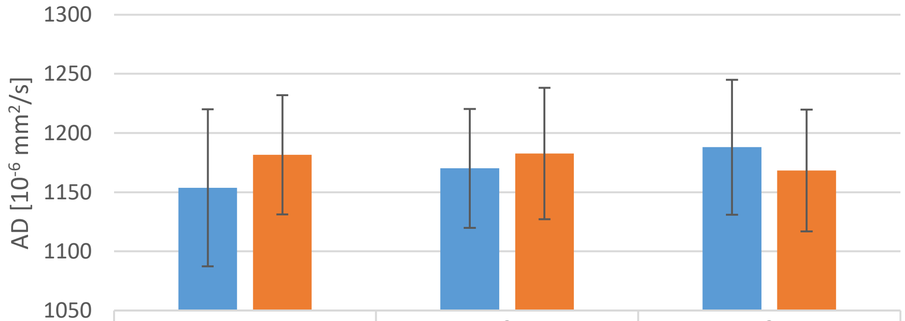

	1	2	3
CON	1154	1170	1188
HTN	1182	1183	1168

L7 – Superior longitudinal fasciculus left

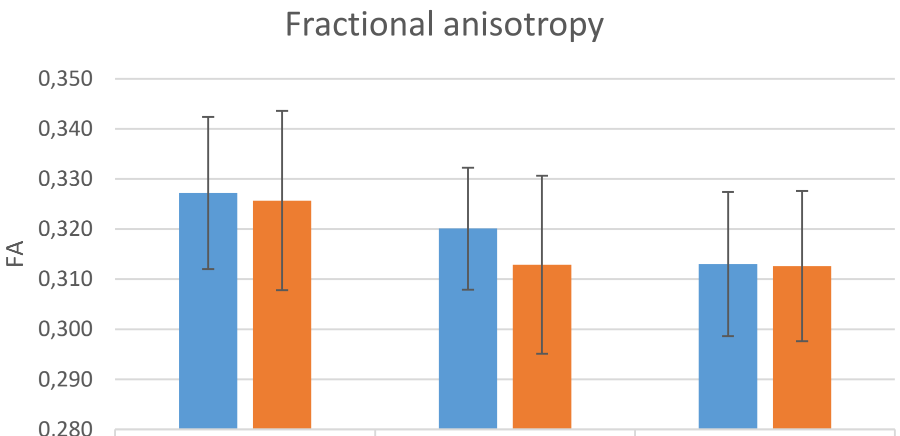

	1	2	3
CON	0,327	0,320	0,313
HTN	0,326	0,313	0,313

***

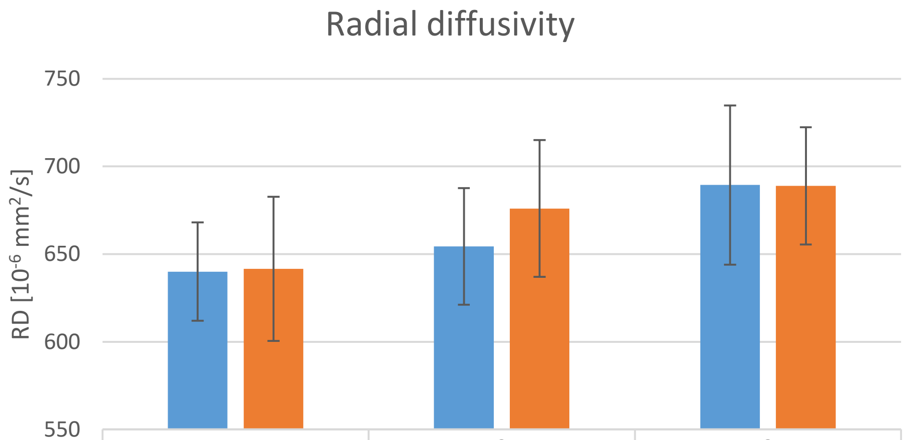

	1	2	3
CON	640	654	689
HTN	642	676	689

***

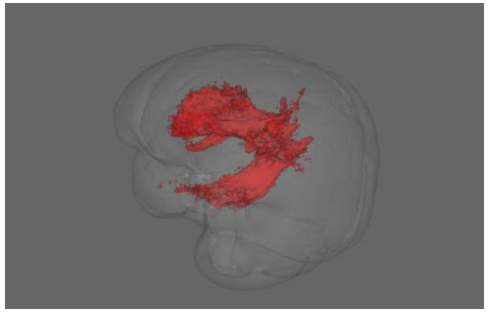

Control group

Patients with hypertension

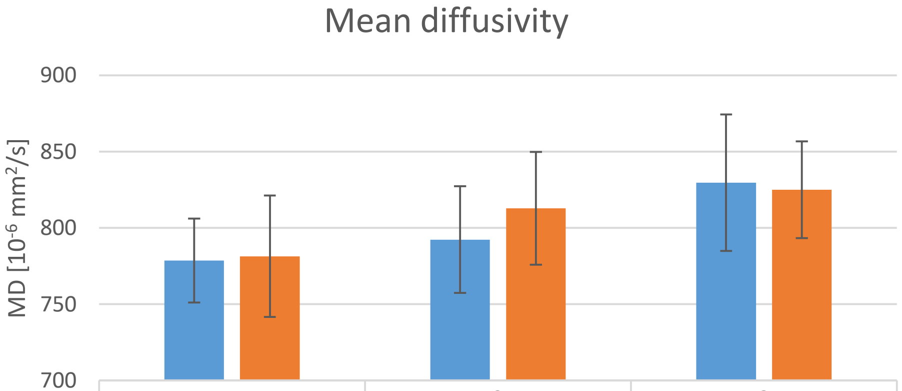

	1	2	3
CON	779	792	830
HTN	781	813	825

***

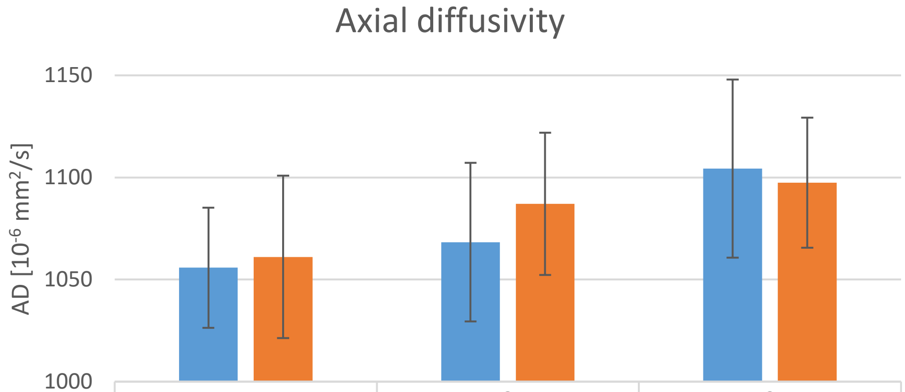

	1	2	3
CON	1056	1068	1104
HTN	1061	1087	1097

***

R7 – Superior longitudinal fasciculus right

Fractional anisotropy

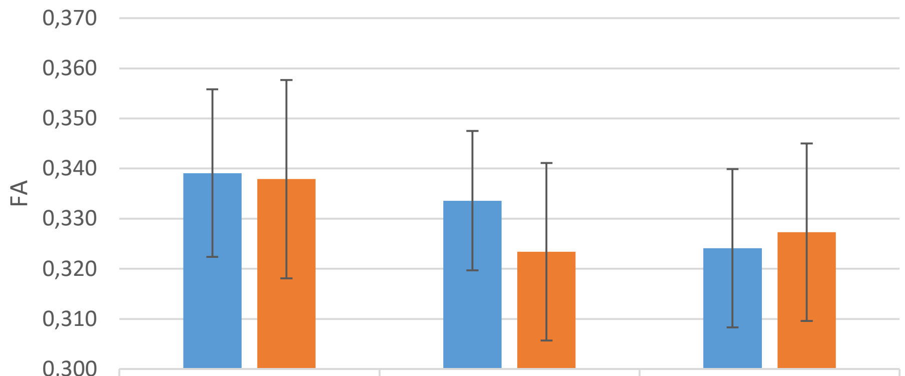

	1	2	3
CON	0,339	0,334	0,324
HTN	0,338	0,323	0,327

**

Mean diffusivity

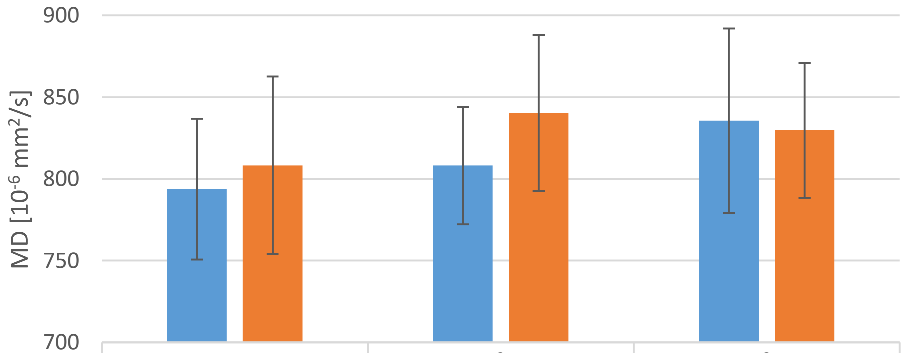

	1	2	3
CON	794	808	836
HTN	808	840	830

**

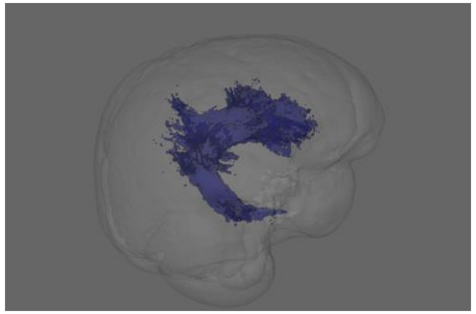

Control group

Patients with hypertension

Radial diffusivity

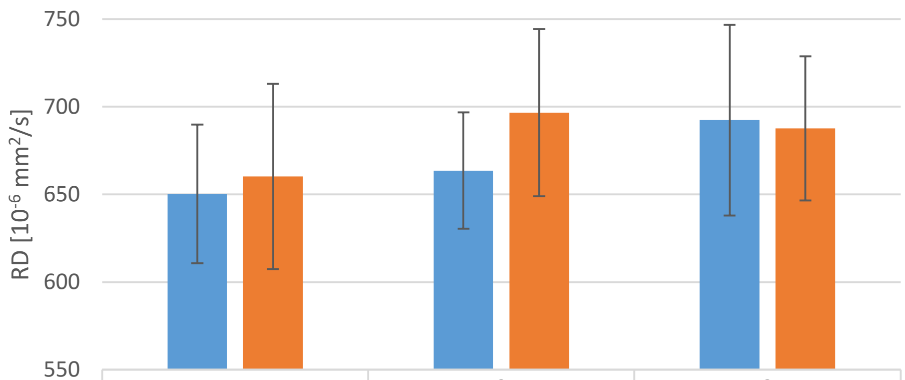

	1	2	3
CON	650	664	692
HTN	660	697	688

**

Axial diffusivity

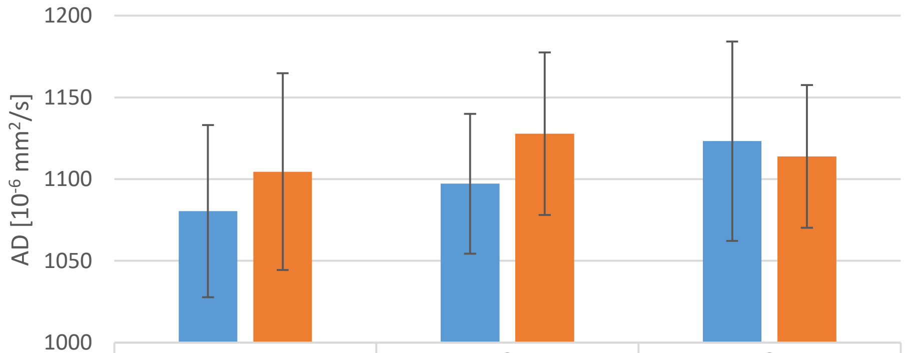

	1	2	3
CON	1080	1097	1123
HTN	1105	1128	1114

*

L8 – Superior longitudinal fasciculus (temporal part) left

Fractional anisotropy

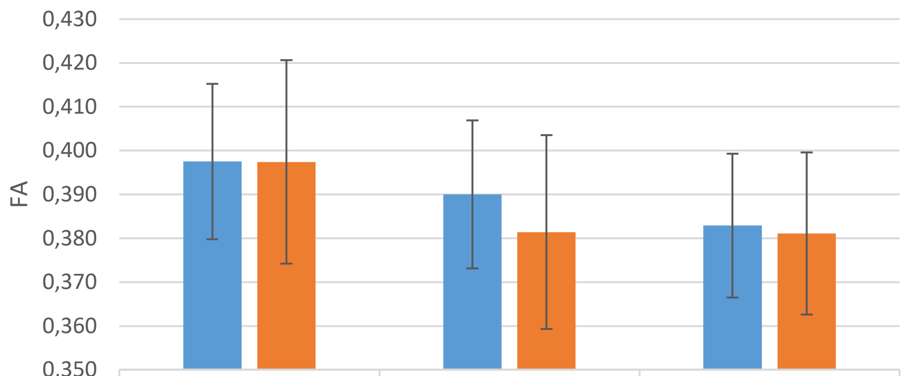

	1	2	3
CON	0,398	0,390	0,383
HTN	0,397	0,381	0,381

**

Radial diffusivity

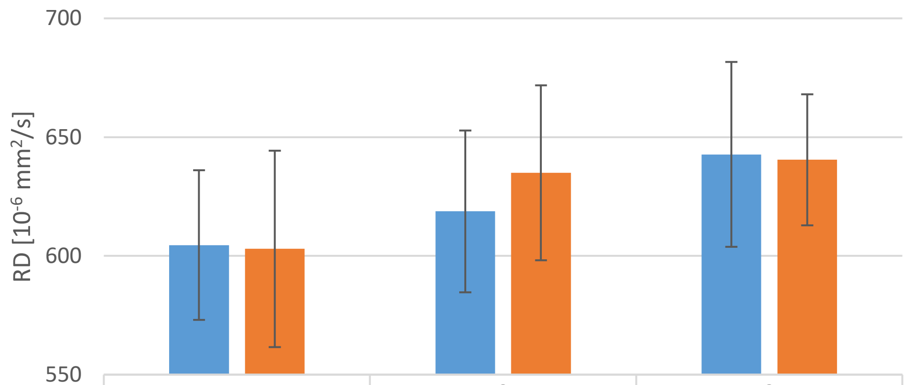

	1	2	3
CON	605	619	643
HTN	603	635	640

***

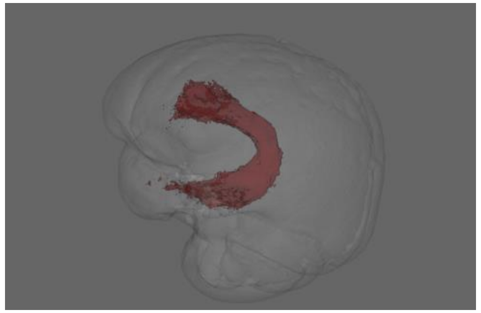

Control group

Patients with hypertension

Mean diffusivity

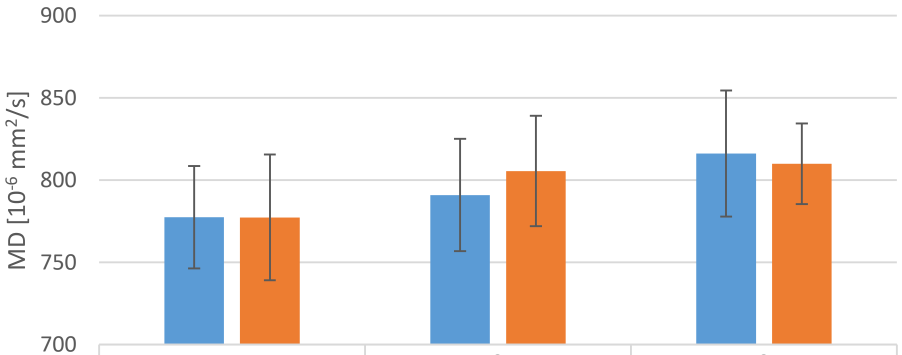

	1	2	3
CON	778	791	816
HTN	777	806	810

***

Axial diffusivity

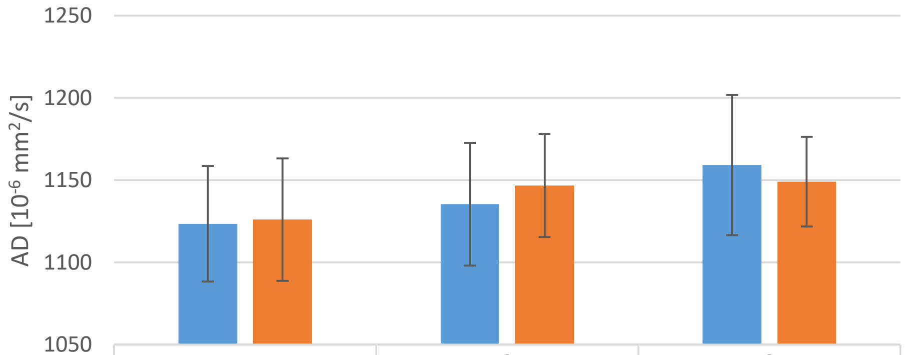

	1	2	3
CON	1123	1135	1159
HTN	1126	1147	1149

***

R8 – Superior longitudinal fasciculus (temporal part) right

Fractional anisotropy

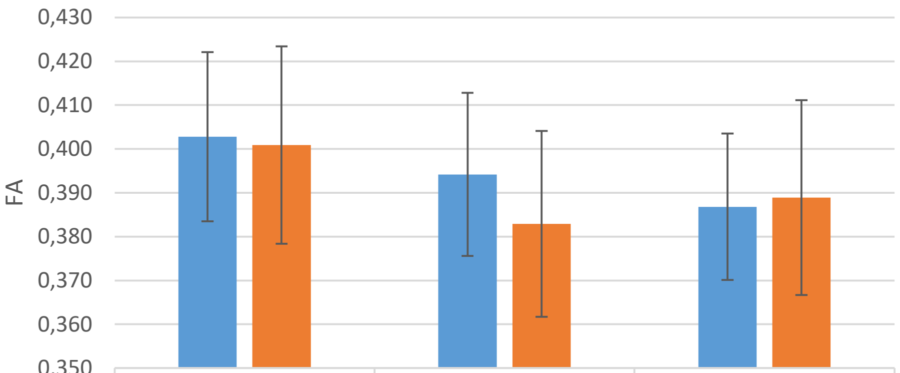

	1	2	3
CON	0,403	0,394	0,387
HTN	0,401	0,383	0,389

**

Radial diffusivity

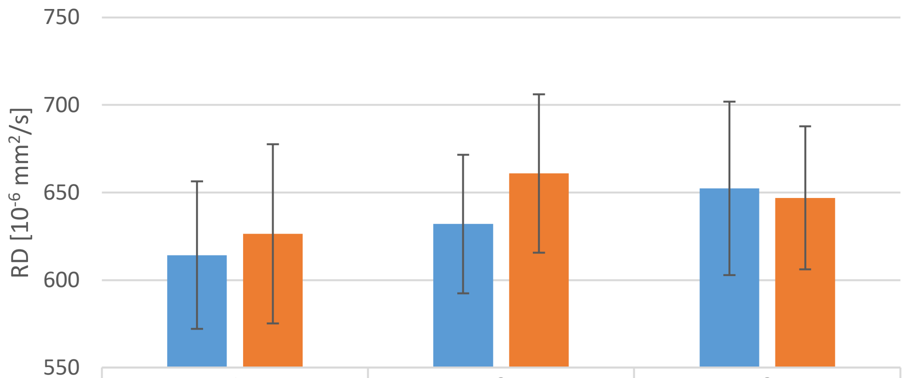

	1	2	3
CON	614	632	652
HTN	626	661	647

**

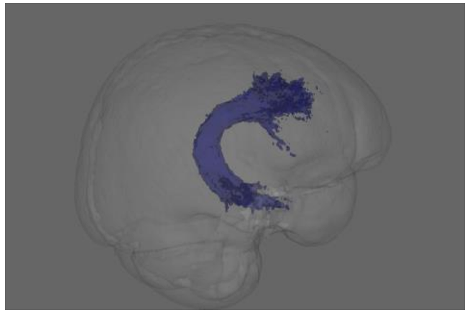

Control group

Patients with hypertension

Mean diffusivity

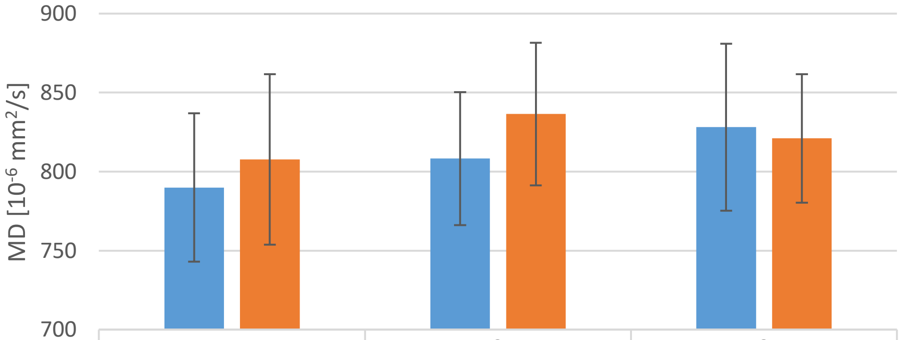

	1	2	3
CON	790	808	828
HTN	808	836	821

*

Axial diffusivity

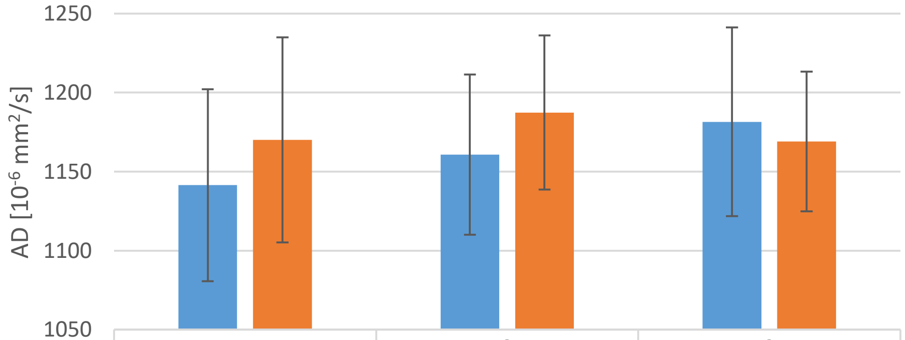

	1	2	3
CON	1141	1161	1181
HTN	1170	1187	1169

L9 – Uncinate fasciculus left

Fractional anisotropy

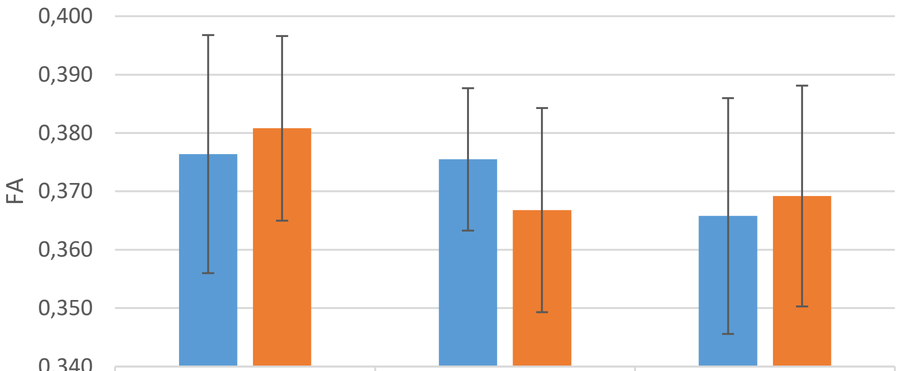

	1	2	3
CON	0,376	0,376	0,366
HTN	0,381	0,367	0,369

*

Radial diffusivity

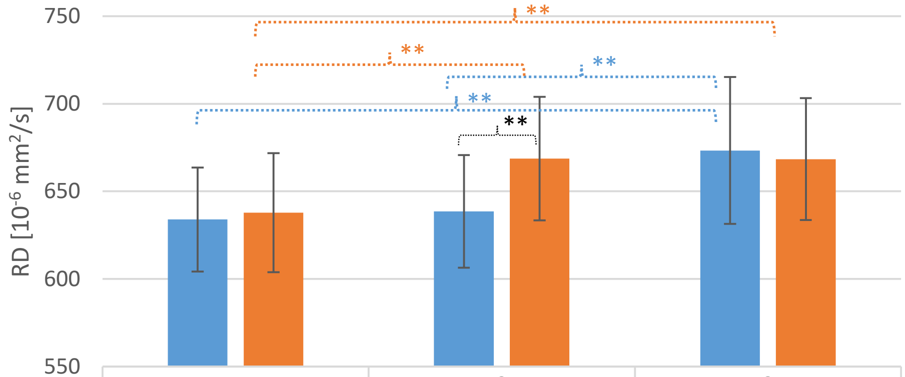

	1	2	3
CON	634	639	673
HTN	638	669	668

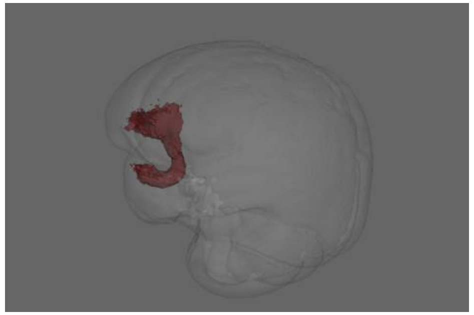

Control group

Patients with hypertension

Mean diffusivity

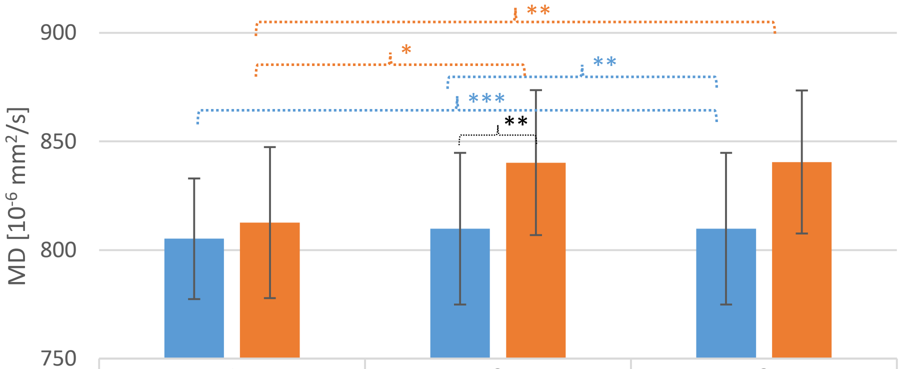

	1	2	3
CON	805	810	810
HTN	813	840	841

Axial diffusivity

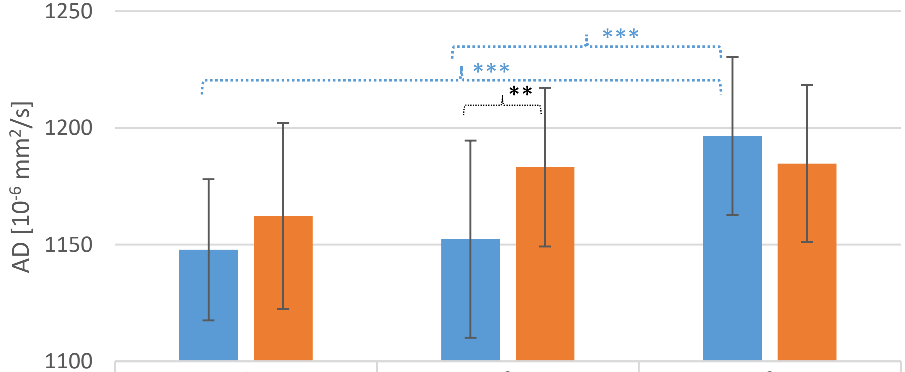

	1	2	3
CON	1148	1152	1197
HTN	1162	1183	1185

R9 – Uncinate fasciculus right

Fractional anisotropy

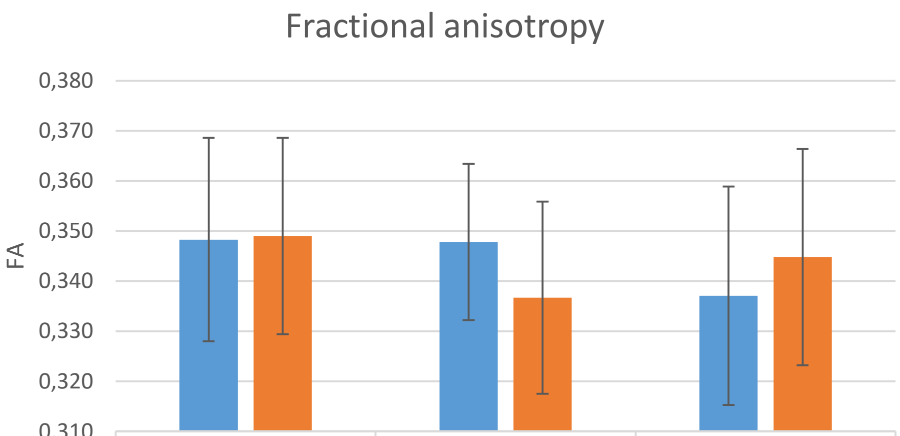

	1	2	3
CON	0,348	0,348	0,337
HTN	0,349	0,337	0,345

Radial diffusivity

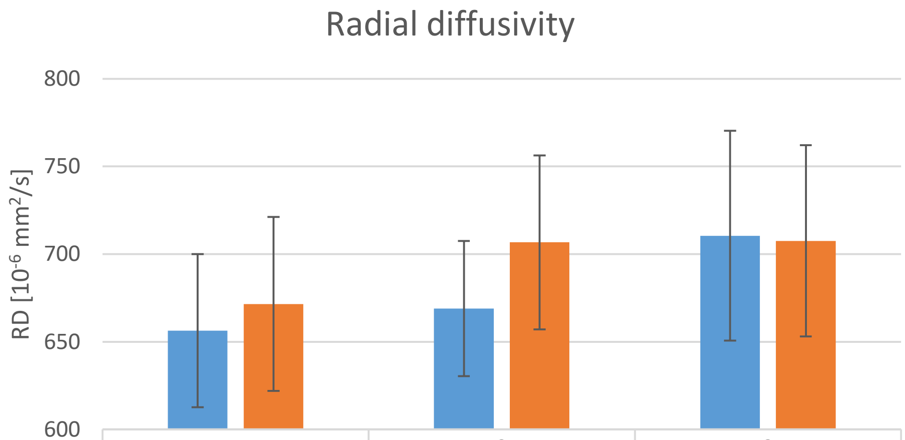

	1	2	3
CON	656	669	710
HTN	672	707	708

* (CON vs HTN)

***

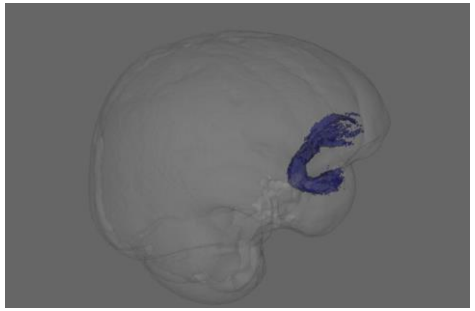

Control group

Patients with hypertension

Mean diffusivity

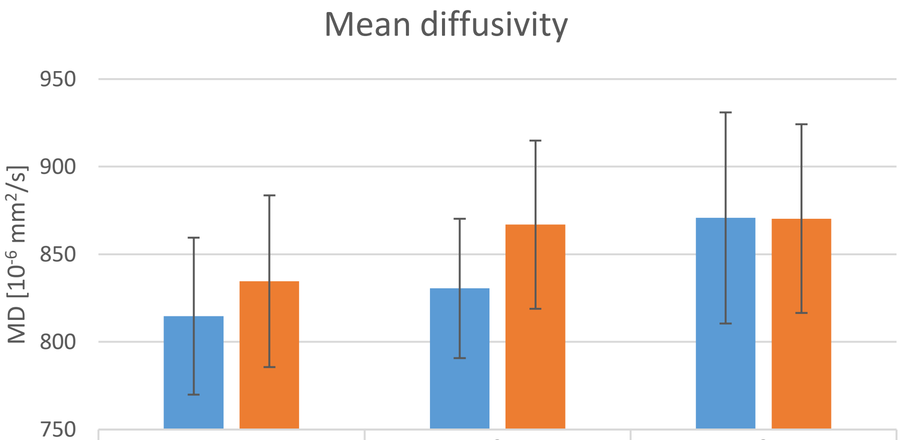

	1	2	3
CON	815	831	871
HTN	835	867	870

* (CON vs HTN)

***

Axial diffusivity

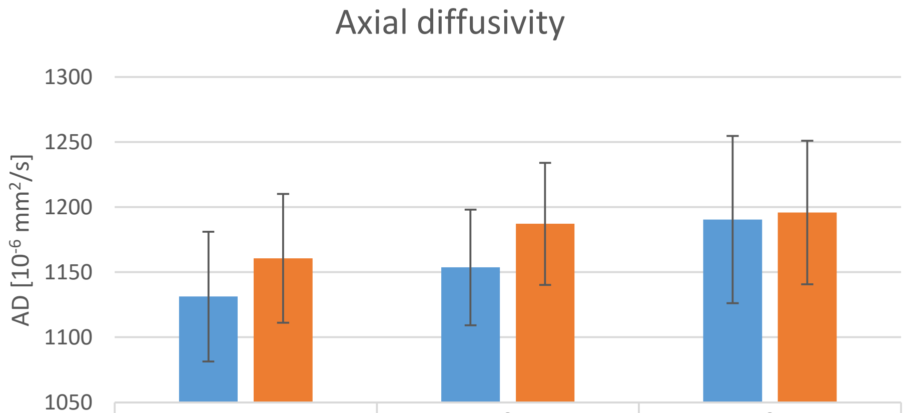

	1	2	3
CON	1131	1154	1190
HTN	1161	1187	1196

* (CON vs HTN)

***
